# Increased SARS-CoV-2 IgG4 has variable consequences dependent upon Fc function, Fc receptor polymorphism, and viral variant

**DOI:** 10.1126/sciadv.ads1482

**Published:** 2025-02-26

**Authors:** L. Carissa Aurelia, Ruth A. Purcell, Robert M. Theisen, Andrew Kelly, Robyn Esterbauer, Pradhipa Ramanathan, Wen Shi Lee, Bruce D. Wines, P. Mark Hogarth, Jennifer A. Juno, Lilith F. Allen, Katherine A. Bond, Deborah A. Williamson, Janine M. Trevillyan, Jason A. Trubiano, Thi HO Nguyen, Katherine Kedzierska, Adam K. Wheatley, Stephen J. Kent, Kelly B. Arnold, Kevin John Selva, Amy W. Chung

**Affiliations:** ^1^ Department of Microbiology and Immunology, Peter Doherty Institute for Infection and Immunity, University of Melbourne, Melbourne, VIC, Australia.; ^2^ Department of Biomedical Engineering, University of Michigan, Ann Arbor, MI, United States.; ^3^ Immune Therapies Group, Burnet Institute, Melbourne, VIC, Australia.; ^4^ Department of Clinical Pathology, University of Melbourne, Melbourne, VIC, Australia.; ^5^ Department of Immunology and Pathology, Central Clinical School, Monash University, Melbourne, VIC, Australia.; ^6^ Victorian Infectious Disease Reference Laboratory (VIDRL), at the Peter Doherty Institute for Infection and Immunity, Melbourne, VIC, Australia.; ^7^ Department of Microbiology, Royal Melbourne Hospital, Melbourne, VIC, Australia.; ^8^ Department of Infectious Diseases, Peter Doherty Institute for Infection and Immunity, University of Melbourne, Melbourne, VIC, Australia.; ^9^ School of Medicine, University of St Andrews, Fife KY16 9TF, Scotland.; ^10^ Department of Infectious Diseases and Immunology, Austin Health, Heidelberg, VIC, Australia.; ^11^ Centre for Antibiotic Allergy and Research, National Centre for Infections in Cancer, Peter MacCallum Cancer Centre, Melbourne, VIC, Australia.; ^12^ Melbourne Sexual Health Centre and Department of Infectious Diseases, Alfred Health, Central Clinical School, Monash University, Melbourne, VIC, Australia.

## Abstract

Repeated mRNA COVID-19 vaccination increases spike-specific immunoglobulin G4 (IgG4) titers. Here, we characterized the influence of increased IgG4 titers on a range of Fc-mediated responses. Elevated spike-specific IgG4 reduced binding to FcγRIIIa and decreased antibody-dependent cellular cytotoxicity. However, in individuals with lower total spike-specific IgG, IgG4 acted in synergy with other IgG subclasses to improve FcγRI and FcγRIIa binding and consequently antibody-dependent cellular phagocytosis. Furthermore, this trend was more pronounced with more recent SARS-CoV-2 variants where vaccination induced comparably lower total spike-specific titers. These observations were further confirmed by in silico modeling where antibody subclass concentrations and FcγR polymorphisms were modulated. Collectively, we illustrate that the impact of elevated IgG4 titers upon Fc functions is dependent on multiple interconnected antibody and antigen factors, which should be taken into consideration when dissecting the mechanisms driving an effective Fc-mediated response following vaccination.

## Teaser

Elevated SARS-CoV-2–specific IgG4 titers decrease ADCC but can increase ADCP when spike-specific antibody titers are low.

## INTRODUCTION

Coronavirus disease 2019 (COVID-19) vaccination elicits high titers of spike-specific antibodies. These antibodies can provide protection through direct neutralization of the virus (*1*) along with the coordination of innate immune responses via the crystallizable (Fc) region (*2*–*4*). These Fc effector functions are induced by severe acute respiratory syndrome coronavirus 2 (SARS-CoV-2)–specific immunoglobulin G (IgG) antibodies, which form immune complexes with Fc gamma receptors (FcγRs) expressed on innate immune cells to activate downstream effector functions such as antibody-dependent cellular cytotoxicity (ADCC) and phagocytosis (ADCP) (*5*). Multiple studies have highlighted the contribution of Fc-mediated responses in providing protection against SARS-CoV-2 (*2*–*4*). A growing number of studies have highlighted the maintenance of Fc effector functions against emerging SARS-CoV-2 variants that evade neutralizing antibodies (*6*–*9*).

The induction of Fc-mediated responses is dependent on the ability of IgG to engage with both its target antigen and the FcγR, both of which are modulated by the biophysical features of the IgG, the FcγR, and the attributes of the target pathogen. This includes the four IgG subclasses (IgG1 to IgG4), which vary in their affinities for FcγRs and ability to activate FcγR-mediated responses (*10*, *11*). Of the four, IgG1 and IgG3 display high capacity to engage with FcγRs, and high titers of these subclasses have been demonstrated to orchestrate polyfunctional antibody responses (*12*, *13*). In contrast, IgG2 and IgG4 are poor inducers of Fc-mediated responses due to their reduced ability to bind activating FcγRs, with several studies suggesting that the presence of IgG2 and IgG4 may impede the Fc effector functions mediated by IgG1 or IgG3 (*12*, *14*, *15*).

In parallel to the four IgG subclasses, multiple classes of FcγRs exist that vary in affinity for IgG subclasses. FcγRI is the sole high-affinity FcγR in humans and can bind all IgG subclasses, except IgG2 (*11*). In contrast, FcγRIIa and FcγRIIIa are low-affinity receptors that are activated through cross-linking by antigen-bound IgG (*5*). Two main FcγRIIa polymorphisms have been identified: FcγRIIa-H131 and FcγRIIa-R131. Similarly, there are two main FcγRIIIa polymorphisms: FcγRIIIa-V158 and FcγRIIIa-F158. FcγRIIa-H131 and FcγRIIIa-V158 display higher affinity for IgG1 and IgG3 compared to FcγRIIa-R131 and FcγRIIIa-F158, respectively (*11*). Moreover, IgG4 can weakly bind FcγRIIIa-V158 but not FcγRIIIa-F158 and has higher affinity for FcγRIIa-R131 compared to FcγRIIa-H131 (*11*). Consequently, Fc functions are dictated by IgG subclass as well as FcγR class and polymorphism.

Intriguingly, multiple studies have reported a rise in spike-specific IgG4 with repeated mRNA vaccination (*16*–*19*). We have previously demonstrated that elevated IgG4 is negatively correlated with FcγR engagement (*16*). Moreover, Irrgang *et al.* (*17*) reported a decline in ADCP accompanying the increase in IgG4, suggesting that elevated IgG4 inhibits Fc effector functions. However, mechanistic studies on the functional consequence of increased IgG4 are now limited especially in regard to ADCC responses and FcγR engagement. Moreover, little is known about how elevated IgG4 influences Fc-mediated responses against SARS-CoV-2 variants.

Here, we demonstrate that IgG4 poorly binds FcγRIIIa, and thus, elevated IgG4 titers impede ADCC. In contrast, IgG4 is capable of mediating moderate ADCP via FcγRI and FcγRIIa. Therefore, a rise in spike-specific IgG4 can compete with other IgG subclasses to impede ADCP, especially when SARS-CoV-2 IgG titers are high. However, when SARS-CoV-2 IgG titers are low, IgG4 works in synergy with other subclasses to enhance ADCP, suggesting that elevated IgG4 titers can be beneficial for ADCP. Furthermore, both experimentally and using in silico models, we demonstrate that in scenarios where SARS-CoV-2 IgG titers are low, such as against emerging novel variants, the presence of elevated IgG4 titers may be beneficial for FcγRI- and FcγRIIa-driven Fc functions. Collectively, our work highlights the complex interplay of multiple features involved in Fc-mediated responses modulated by elevated SARS-CoV-2 IgG4.

## RESULTS

### Increased IgG4 following repeated SARS-CoV-2 mRNA vaccination is negatively correlated with FcγR binding capacity

**Fig. 1.**
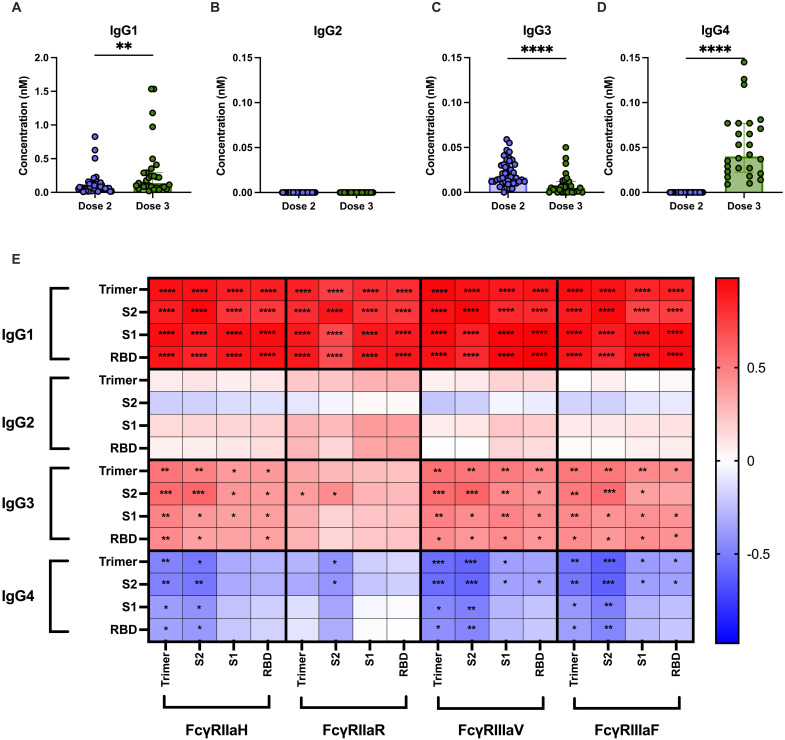
Increased IgG4 following repeated mRNA vaccination is negatively correlated with FcγR immune complex formation. Relative concentration of SARS-CoV-2 ancestral spike trimer-specific (**A**) IgG1, (**B**) IgG2, (**C**) IgG3, and (**D**) IgG4 in plasma collected 1 month (median = 28 days post-vaccination) post-dose two (*n* = 46) and three (*n* = 31) of BNT162b2 vaccine as measured by multiplex. Statistical significance was assessed using Mann-Whitney *U* test. (**E**) Two-tailed Spearman correlation between WT SARS-CoV-2 antigen-specific IgG1 to IgG4 and FcγR (FcγRIIa-H131, FcγRIIa-R131, FcγRIIIa-V158, and FcγRIII-F158) binding. *P < −0.05, **P < 0.01, ***P < 0.001, and ****P < 0.0001. RBD, receptor binding domain.

Plasma samples were collected from SARS-CoV-2 naïve individuals 1 month post-second and post-third dose of mRNA vaccination (table S1). Plasma was assessed for SARS-CoV-2 antigen-specific IgG subclasses (Fig. 1, A to D) using a SARS-CoV-2 multiplex bead assay. A small but significant increase in IgG1 (*P* < 0.01; Fig. 1A) and a modest decrease in IgG3 (*P* < 0.0001; Fig. 1C) were observed between the second and third mRNA doses. In contrast, a significant increase in spike-specific IgG4 (*P* < 0.0001; Fig. 1D) was observed following the third dose.

IgG4 is often associated with reduced Fc functions due to its poor ability to engage with FcγRs (*10*, *20*). Previous studies have demonstrated that elevated IgG4 is accompanied by a reduction in spike-specific Fc-mediated responses (*17*). To confirm this, we measured the ability of post-vaccination plasma to engage with soluble FcγRIIa and FcγRIIIa dimers as a surrogate measurement for ADCP and ADCC, respectively. We did not observe a decrease in FcγR dimer binding following the third dose when testing with equivalent plasma dilutions (1:1600), although a significant decrease in FcγRIIIaV binding was observed when responses were normalized to spike-specific total IgG, in accordance with previous published studies (*P* < 0.0001; fig. S1) (*17*). We further interrogated the relationship between IgG subclasses and FcγR engagement. IgG1 titers against all tested antigens displayed strong positive correlations with binding to all tested FcγRs (FcγRIIa: FcγRIIa-131R or FcγRIIa-131H, FcγRIIIa: FcγRIIIa-158 V or FcγRIIIa-158F), while weaker positive correlations between IgG3 titers and FcγR binding were observed (Fig. 1E). In contrast, IgG4 titers were negatively correlated with FcγR engagement; thus, we next aimed to determine whether elevated IgG4 could potentially reduce FcγR-mediated functions.

### IgG4 inhibits ADCC in a dose-dependent manner

To explore the potential inhibitory role of IgG4, we generated a cocktail of IgG4 monoclonal antibodies (mAbs) consisting of three previously characterized SARS-CoV-2 mAbs, all with high affinity for ancestral SARS-CoV-2 receptor binding domain (RBD) and are of different RBD classes (*21*). We confirmed that subclass switching did not affect antigen binding (figs. S2 and S3 and tables S2 and S3) and that the IgG4 mAbs engaged different FcγR classes and polymorphisms as expected, with overall high affinity to FcγRI similar to IgG1, but weaker binding to FcγRIIa-131H/R and very low or no binding to FcγRIIIa-158 V/F (figs. S2 and S3 and tables S2 and S3).

**Fig. 2.**
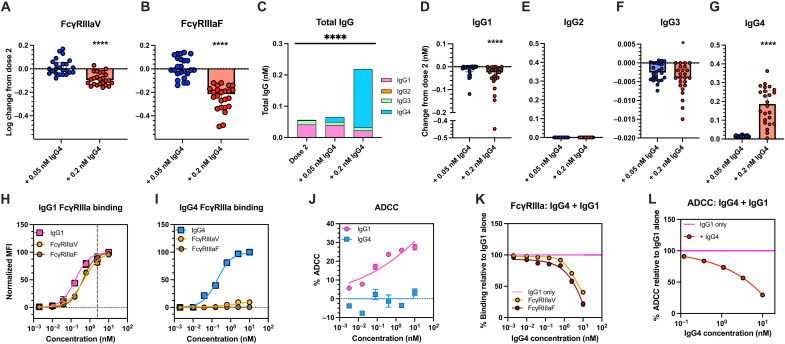
IgG4 reduces FcγRIIIa binding and ADCC in dose-dependent manner. (**A** and **B**) Change in binding of ancestral spike trimer-specific plasma antibodies to soluble FcγRIIIa-V158 and FcγRIIIa-F158 dimers following the addition of IgG4 mAb cocktails into post-dose two plasma. (**C**) Comparison of ancestral spike-specific IgG titers post-dose two BNT162b2 vaccination (*n* = 24) and following addition of 0.05 nM (0.007 μg/ml) or 0.2 nM (0.029 μg/ml) of IgG4 SARS-CoV-2 mAbs. (**D** to **G**) Change in IgG1-IgG4 titers following IgG4 addition into plasma. Significant differences were assessed using Friedman’s test with Dunn’s multiple comparison. Error bars indicate interquartile range. Binding of (**H**) IgG1 and (**I**) IgG4 mAb cocktails to IgG1 or IgG4 detection reagents, FcγRIIIa-V158 and FcγRIIIa-F158 dimers. The normalized median fluorescent intensity (MFI) of each detection reagent was plotted. (**J**) Comparison of the ability of IgG1 and IgG4 mAb cocktails to induce ADCC. (**K**) FcγRIIIaV and FcγRIIIaF binding of IgG4 mAb cocktail into IgG1 mAb cocktail (2.5 nM/0.365 μg/ml). (**L**) ADCC mediated by IgG4 mAb cocktails titrated into IgG1 mAb cocktail (10 nM; 1.46 μg/ml). Error bars indicate SEM. Curves were fitted using a four-parameter nonlinear regression model. ****P < 0.0001.

IgG4 has been described to be a poor inducer of ADCC (*15*, *22*, *23*). To confirm this, we added the IgG4 mAb cocktail into a subset of dose two mRNA vaccinated plasma samples (*n* = 24), mimicking elevated IgG4 observed following dose three mRNA vaccination, and assessed binding to soluble FcγRIIIa dimers. When IgG4 was added to a concentration corresponding to the median concentration observed post-dose three (0.05 nM; 0.007 μg/ml) binding to either FcγRIIIa polymorphism was not significantly different (Fig. 2A) (*11*). As we did not have matched post-dose four vaccination samples, we also performed IgG4 addition at a higher concentration (0.2 nM; 0.029 μg/ml), which corresponded to the median IgG4 concentration of a small subset of post dose four samples (fig. S4). The higher concentration of IgG4 addition significantly decreased FcγRIIIa-158 V/F binding (*P* < 0.0001; Fig. 2B and fig. S5).

To investigate the mechanism behind the differences in response between the two IgG4 concentrations, we assessed antigen-bound IgG1 to IgG4 titers before and after IgG4 addition (Fig. 2, C to G). Spike-bound IgG1 and IgG3 titers were not significantly different following low-concentration IgG4 addition (0.05 nM) and collectively made up most of the antigen-bound IgG (Fig. 2C). In contrast, spike-bound IgG1 and IgG3 were significantly reduced following 0.2 nM addition of IgG4 (IgG1: *P* < 0.0001; IgG3: *P* < 0.05; Fig. 2, D and F, and fig. S5), suggesting that high concentrations of IgG4 may be outcompeting other subclasses for antigen.

To confirm that elevated IgG4 reduces ADCC responses, we performed a previously described primary natural killer (NK) cell ADCC assay against spike-expressing target cells (*24*). The IgG1 mAbs induced potent ADCC and formed immune complexes with soluble FcγRIIIa-158 V/F (Fig. 2, H and J, and fig. S3), whereas the IgG4 mAbs was unable to induce ADCC above background levels and displayed poor and no measurable binding to FcγRIIIa-158 V and FcγRIIIa-158F, respectively (Fig. 2, I and J, and fig. S3). Last, titrating the IgG4 mAb cocktail into the IgG1 mAb cocktail demonstrated that IgG4 reduced FcγRIIIa binding and ADCC in a concentration-dependent manner (Fig. 2, K and L). Collectively, these results demonstrate that excessive IgG4 can inhibit FcγRIIIa binding and ADCC.

### Elevated IgG4 titers can enhance ADCP when total SARS-CoV-2 titers are low

**Fig. 3.**
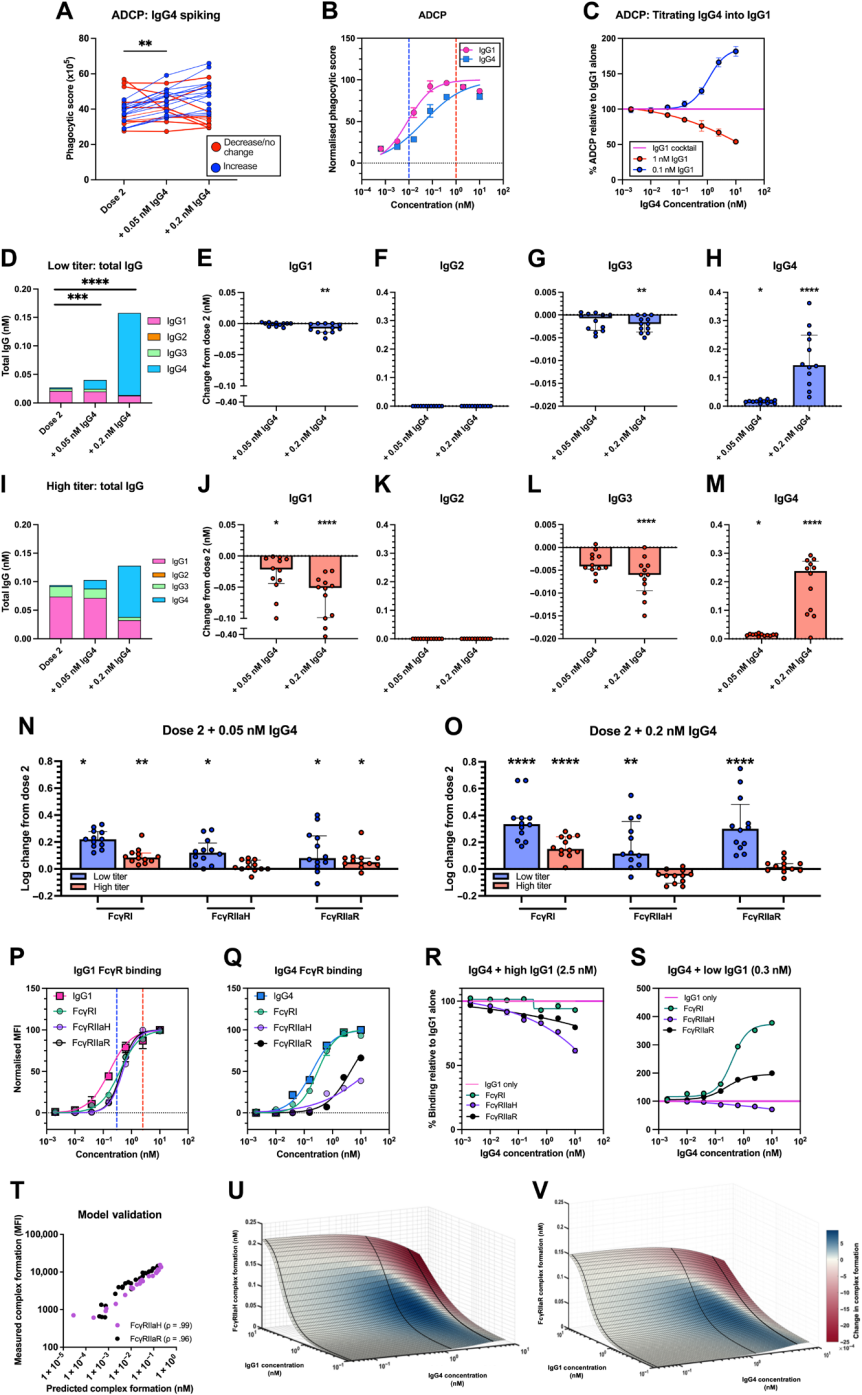
Change in ADCP and FcγR engagement following IgG4 addition into post second dose BNT162b2 plasma. (**A**) ADCP activity of plasma collected post-dose two of BNT162b2 vaccination (*n* = 24) before and after addition of IgG4 SARS-CoV-2 mAb cocktail. (**B**) ADCP activity induced by IgG1 (pink) and IgG4 (blue) mAb cocktails. (**C**) ADCP of ancestral spike-coated beads mediated by IgG4 mAb cocktail titrated into IgG1 mAb cocktail at 1 nM (0.146 μg/ml; red) or 0.1 nM (0.015 μg/ml; blue). Change in total IgG and IgG subclass distribution of spike-bound antibodies in individuals with (**D** to **H**) low total IgG (below median IgG titer) and (**I** to **M**) high total IgG (above median IgG titer) post-dose two and following addition IgG4. (**N** and **O**) Log change in FcγRI, FcγRIIa-H131, and FcγRIIa-R131 binding following (N) 0.05 nM (0.007 μg/ml) and (O) 0.2 nM (0.029 μg/ml) IgG4 addition into post-dose two plasma. Significant differences were assessed using Friedman’s test with Dunn’s multiple comparison. Binding of (**P**) IgG1 and (**Q**) IgG4 mAb cocktails to IgG1 or IgG4 detection reagents and recombinant soluble FcγRI, FcγRIIa-H131, and FcγRIIa-R131. The normalized MFI of each detection reagent was plotted. (**R** and **S**) FcγRI and FcγRIIa binding of IgG4 mAb cocktail titrated into IgG1 mAb cocktail at (R) saturating (2.5 nM/0.365 μg/ml) or (S) half-maximal binding (EC_50_; 0.3 nM/0.044 μg/ml) IgG1 concentration and binding to FcγRs were assessed. Curves were fitted using a four-parameter nonlinear regression model. (**T**) Model predictions for FcγRIIa-H131 and FcγRIIa-R131 immune complex formation were compared with multiplex experimental measurements. Landscape illustrating the relationship between IgG1 and IgG4 concentrations for predicting (**U**) FcγRIIa-H131 and (**V**) and FcγRIIa-R131 immune complex formation. Colour indicated the predicted change in complex formation from baseline (blue, decrease; red, increase). *P < 0.05, **P < 0.01, ***P < 0.001, and ****P < 0.0001.

We next investigated the influence of elevated levels of IgG4 upon ADCP by adding the IgG4 cocktail to dose two mRNA-vaccinated plasma samples using a previously described SARS-CoV-2 bead-based ADCP assay (*24*, *25*). Unexpectedly, we observed that 67% of vaccinees displayed improved ADCP (>10% increase in phagocytic score; Fig. 3A, blue lines) (*P* < 0.01). We confirmed this was mediated via a FcR-dependent manner by repeating the IgG4 spiking in the presence of an Fc block, which did not result in phagocytosis above background (fig. S6B). Given that these results contrasted with previous findings suggesting that IgG4 inhibits ADCP induction (*17*), we examined the ability of the IgG4 mAb cocktails alone to induce ADCP. We found that the IgG4 mAbs were capable of mediating moderate ADCP, albeit reduced compared to IgG1 [IgG1 median effective concentration (EC_50_): 0.009 nM versus IgG4 EC50: 0.044 nM; Fig. 3B]. Therefore, we hypothesized that IgG4 addition into plasma may enhance ADCP by working in synergy with other SARS-CoV-2 antibodies when total titers are low. Whereas when SARS-CoV-2 antibody titers are high, the addition of IgG4 may sterically hinder or outcompete IgG1 for antigen and/or FcγR engagement, thus reducing ADCP.

To demonstrate this, we titrated the IgG4 mAbs into a fixed high concentration of IgG1 mAb cocktail (1 nM; 0.146 μg/ml) and assessed for ADCP. The addition of IgG4 reduced IgG1-mediated ADCP (29% reduction at 1:1 IgG1:IgG4 ratio; Fig. 3C, red line), demonstrating that IgG4 can inhibit ADCP. In contrast, titrating IgG4 into a lower concentration of IgG1 (0.1 nM; 0.015 μg/ml) enhanced ADCP (80% enhancement at the highest concentration of IgG4 added; Fig. 3C, blue line). Collectively, our results propose two different consequences of elevated IgG4 upon ADCP depending on total SARS-CoV-2 IgG titers. When IgG titers are high, IgG4 competes with other subclasses for antigen and/or FcγR binding, leading to a reduction of ADCP. However, when titers are low, increased IgG4 results in more antigen-bound antibody, leading to an overall increase in ADCP.

To further investigate the role of total spike-specific IgG titers upon ADCP responses, we divided our cohort based on their SARS-CoV-2 spike-specific IgG concentrations post-second dose mRNA vaccination (low/high spike-specific total titers) and examined subclass composition of their spike-specific IgG before and after IgG4 addition (Fig. 3, D to M, and fig. S5). In the low titer group, low concentration IgG4 addition did not significantly change antigen engagement by any subclass, except for IgG4 (Fig. 3, D to H). In contrast, low concentration IgG4 addition caused a significant reduction in spike-bound IgG1 in the high titer group (*P* < 0.05; Fig. 3J), and a trend toward reduced IgG3, albeit nonsignificant (Fig. 3L). This suggests IgG4 competes with other subclasses for antigen binding when spike-specific titers are high, but this does not occur when titers are low.

As THP-1 monocytes express both FcγRI and FcγRII (fig. S6D), we assessed the impact of IgG4 addition on antigen-specific antibody engagement to soluble FcγRI and FcγRIIa. IgG4 spiking resulted in an overall increase in FcγRI binding at both tested concentrations (0.05 nM IgG4, *P* < 0.05; 0.2 nM IgG4, *P* < 0.0001; Fig. 3, N and O, and fig. S5). IgG4 addition resulted in heterogenous changes in FcγRIIa engagement. In the low titer group, FcγRIIaH and FcγRIIaR binding improved following IgG4 addition at both concentrations (1.2-fold increase, *P* < 0.05; Fig. 3, N and O, and fig. S5), suggesting that IgG4 is working in synergy with other subclasses to improve FcγRIIa binding. Changes in FcγRIIa binding varied in the high titer group depending on the polymorphism and concentration of IgG4 added. Binding to FcγRIIaH did not change regardless of the concentration of IgG4 added. In contrast, the high titer group had improved FcγRIIaR engagement following low concentration IgG4 addition (1.1-fold, *P* < 0.05; Fig. 3N), but spiking of IgG4 at high concentrations did not result in any change in FcγRIIaR binding (Fig. 3, N and O). Collectively, these results suggests that presence of low IgG4 can enhance overall FcγRI and FcγRIIaR binding, especially when titers are low. Furthermore, our results highlight how IgG4 interacts differently with the two FcγRIIa polymorphisms.

To gain a better understanding of the mechanism behind the induction and/or inhibition of FcγRI and FcγRIIa binding by IgG4, we used the mAb cocktails to mimic the different vaccine scenarios. We first confirmed the ability of the IgG1 and IgG4 mAb cocktails to form FcγRI and FcγRIIa immune complexes. As expected, IgG4 displayed lower binding to FcγRIIaH and FcγRIIaR compared to IgG1 (FcγRIIaR EC_50_ IgG1: 0.49 nM versus IgG4: 4.3 nM; FcγRIIaH EC_50_ IgG1: 0.49 nM versus IgG4: 19.3 nM; Fig. 3, P and Q, and table S3), but both subclasses exhibited similar capacity to bind FcγRI (IgG1 EC_50_: 0.39 nM; IgG4 EC_50_: 0.30 nM; Fig. 3, P and Q). Similar to previous reports (*11*), we observed higher binding of IgG4 to FcγRIIaR than FcγRIIaH (Fig. 3Q), resulting in smaller differences in binding between IgG1 and IgG4 for FcγRIIaR than for FcγRIIaH (FcγRIIaR EC_50_ IgG1: 0.49 nM versus IgG4: 4.3 nM; FcγRIIaH EC_50_ IgG1: 0.49 nM versus IgG4: 19.3 nM), further supporting the differences that were observed between the two polymorphisms upon IgG4 addition to dose two plasma samples.

To further interrogate how the relationship between IgG4 and other subclasses influences FcγRI and FcγRIIa binding, we titrated IgG4 into a high fixed concentration of IgG1 (2.5 nM, 0.37 μg/ml). IgG4 addition had minimal impact on FcγRI binding Fig. 3R). In contrast, binding to both FcγRIIa polymorphisms was reduced (Fig. 3R); however, a greater reduction was seen for FcγRIIaH compared to FcγRIIaR (FcγRIIaH: 24% reduction; FcγRIIaR: 12% reduction at 1:1 IgG1:IgG4 ratio).

Last, we titrated the IgG4 cocktail into the IgG1 cocktail at approximately EC_50_ concentration (0.3 nM, 0.04 μg/ml). At this concentration, IgG4 addition improved FcγRI and FcγRIIaR complex formation (FcγRI: 60% increase, FcγRIIaR: 35% increase; Fig. 3S). However, FcγRIIaH was reduced when excess IgG4 was added (19% reduction at 8:1 IgG4:IgG1 ratio) but had minimal effect (<10% difference in binding) when IgG4 was added at lower concentrations (Fig. 3S), mirroring the changes in FcγR binding observed following IgG4 addition into post-dose two plasma.

### In silico models confirm that IgG4 can enhance FcγRIIa binding when IgG1 titers are low but inhibits Fc functions at high IgG1 titers

To gain a further mechanistic understanding of the importance of spike-specific IgG titers relative to IgG4 titers in influencing FcγRIIa immune complex formation, we applied a qualitative computational framework that utilizes a previously developed ordinary differential equation (ODE) model that predicts Fc effector functions following different IgG subclass distributions upon HIV vaccination (*26*, *27*). First, we validated that the computational model accurately predicted FcγRIIaH (Pearson ρ = 0.99) and FcγRIIaH (ρ = 0.96) relative to experimental FcγRIIa multiplex measurements (Fig. 3T). We then used the model to predict FcγRIIa immune complex formation as spike-specific IgG1 (the most abundant subclass), and IgG4 concentrations were simultaneously altered to reflect increases that may result from boosting (Fig. 3, U and V). The resulting landscape illustrates that at lower IgG1 concentrations, the addition of low to moderate levels of IgG4 induce an upward incline in FcγRIIa (Fig. 3, U and V, blue regions), whereas the high addition of IgG4 even to lower concentrations of IgG1 resulted in a decreased change in FcγRIIa immune complex formation, with the greatest decrease occurring when simultaneous high levels of IgG1 and IgG4 concentrations are present (Fig. 3, U and V, red regions). Model dissection also confirmed that this mechanism is driven by changing levels of competition between IgG1 and IgG4 depending on IgG1 levels. Overall, this simulation provides quantitative confirmation for a dual role of IgG4, whereby it can either enhance or reduce ADCP, depending on an individual’s original spike-specific IgG1 titers.

### IgG4 depletion confirms IgG4 can outcompete IgG1 and IgG3 for antigen binding and reduce FcγRIIIa binding

**Fig. 4.**
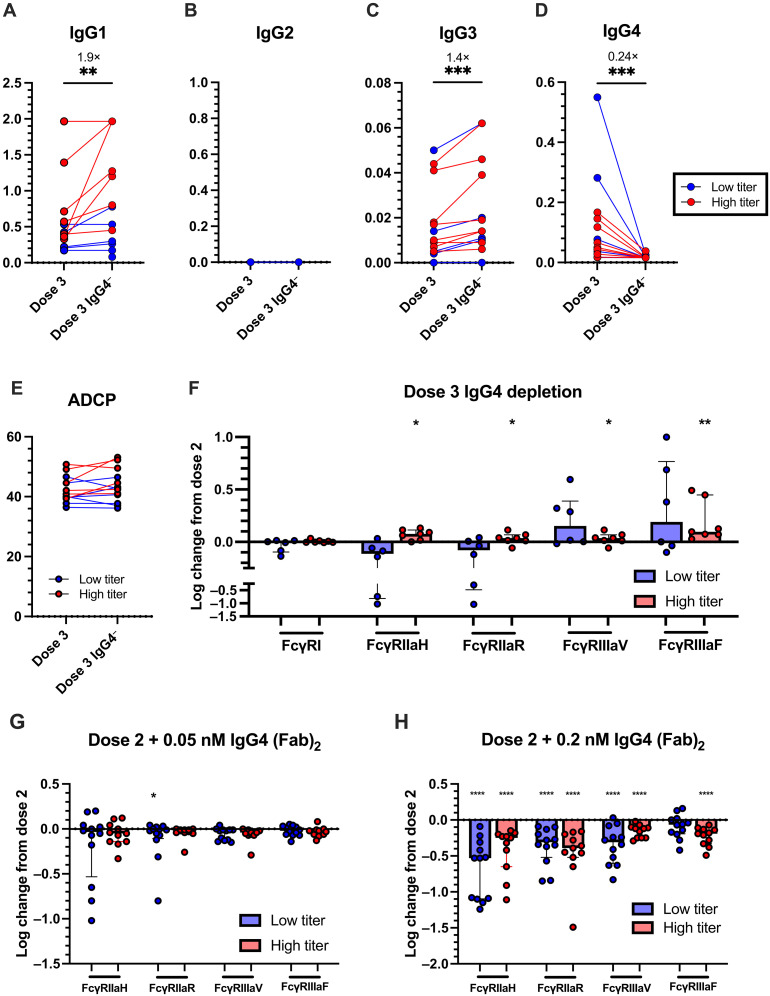
IgG4 depletion and (Fab′)_2_ spiking confirms dual role of IgG4. Comparison of (**A** to **D**) spike-bound IgG1 to IgG4 titers and (**E**) ADCP responses of plasma collected post dose three mRNA vaccination (*n* = 13, median = 28 days post-vaccination) and IgG4 depleted plasma. (**F**) Log change in binding to soluble FcγRs of post-dose three plasma following IgG4 depletion. Statistical significance was assessed using Wilcoxon test. Log change in binding to FcγRIIa and FcγRIIIa of post-dose two mRNA vaccination plasma (*n* = 24, median = 28 days post-vaccination) following (**G**) 0.05 nM (0.006 μg/ml) and (**H**) 0.2 nM (0.022 μg/ml) addition of (Fab)_2_ fragments of IgG4 mAbs into. Significant differences were assessed using Friedman’s test with Dunn’s multiple comparison. *P < 0.05, **P < 0.01, ***P < 0.001, and ****P < 0.0001.

Although the above IgG4 spiking assays suggest that IgG4 is detrimental for FcγRIIIa-mediated responses but may enhance FcγRIIa-mediated responses, we sought to characterize a more definitive role of IgG4 by depleting IgG4 from a subset of post-dose three plasma (*n* = 16). We confirmed that IgG4 was depleted by >75% in all samples [median 96%, interquartile range (IQR) 89 to 98%]. We next compared antigen affinity of bulk and IgG4-depleted plasma by biolayer interferometry (BLI). Given that plasma consists of polyclonal pools of antibodies, which are present at various concentrations, a dissociation constant equilibrium (*K*_d_) is difficult to accurately calculate. Therefore, only the dissociation rate constant (*k*_dis_), which is a concentration-independent measurement, was measured (*28*, *29*). We observed similar binding disassociation for both IgG4 depleted and undepleted plasma (fig. S7), suggesting that IgG4 had similar affinity to spike as other subclasses. We next investigated the contribution of IgG4 to Fc effector functions. The depletion of IgG4 allowed for other IgG subclasses to bind antigen, resulting in significantly higher spike-bound IgG1 and IgG3 (IgG1: 1.9-fold increase, *P* < 0.01; IgG3: 1.4-fold increase, *P* < 0.001; Fig. 4, A and C). This also confirms that polyclonal spike-specific IgG4 can compete with IgG1 and IgG3 for antigen binding.

We next investigated the contribution of IgG4 to Fc effector functions by assessing ADCP responses and FcγR binding. As with the spiking assays, we divided our cohort based on their total IgG titers post-IgG4 depletions. ADCP responses between IgG4-depleted and -undepleted plasma was not significantly different, regardless of total IgG titers (Fig. 4E). Likewise, no differences in FcγRI binding were observed between IgG-depleted and -undepleted plasma (Fig. 4F), likely due to the comparable affinity of IgG1 and IgG4 for FcγRI. Similar to IgG4 spiking, IgG4 depletion resulted in heterogenous changes to FcγRIIa binding (Fig. 4E). We found that individuals with high total IgG titers following depletion displayed improved FcγRIIa binding upon IgG4 depletion (*P* < 0.05). In contrast, individuals with low IgG titers showed a trend of reduced FcγRIIa binding following depletion, supporting previous observations that IgG4 can work in synergy with other subclasses to improve FcγRIIa binding. Last, we observed a significant increase in FcγRIIIa binding post-IgG4 depletion in the high titer group (*P* < 0.05) and a trend toward increased FcγRIIIa binding post-depletion in the low titer group, confirming that elevated IgG4 inhibits FcγRIIIa binding.

### (Fab′)_2_ spiking confirms outcompetition of IgG1 and IgG3 reduces FcγR-mediated responses

To confirm that competition for antigen binding was driving reduced FcγR-mediated responses, we repeated the IgG4 mAb spiking assays using the (Fab′)_2_ fragments of the IgG4 mAbs. As with IgG4 spiking, (Fab′)_2_ spiking reduced spike binding of IgG1 and IgG3 (fig. S8B). Low-concentration (Fab′)_2_ spiking either resulted in a decreases or no change in FcγR binding regardless of IgG titers binding in individuals with low IgG (Fig. 4F). High-concentration (Fab′)_2_ spiking significantly decreased binding to FcγRIIa and FcγRIIIa (*P* < 0.0001; Fig. 4G). Collectively, this confirms that outcompetition of IgG1 and IgG3 for antigen binding decreases FcγR engagement and that the Fc region of IgG4 drives the observed increase in FcγRIIa engagement.

### IgG4 addition improves formation of FcγRIIa immune complexes against SARS-CoV-2 variants

**Fig. 5.**
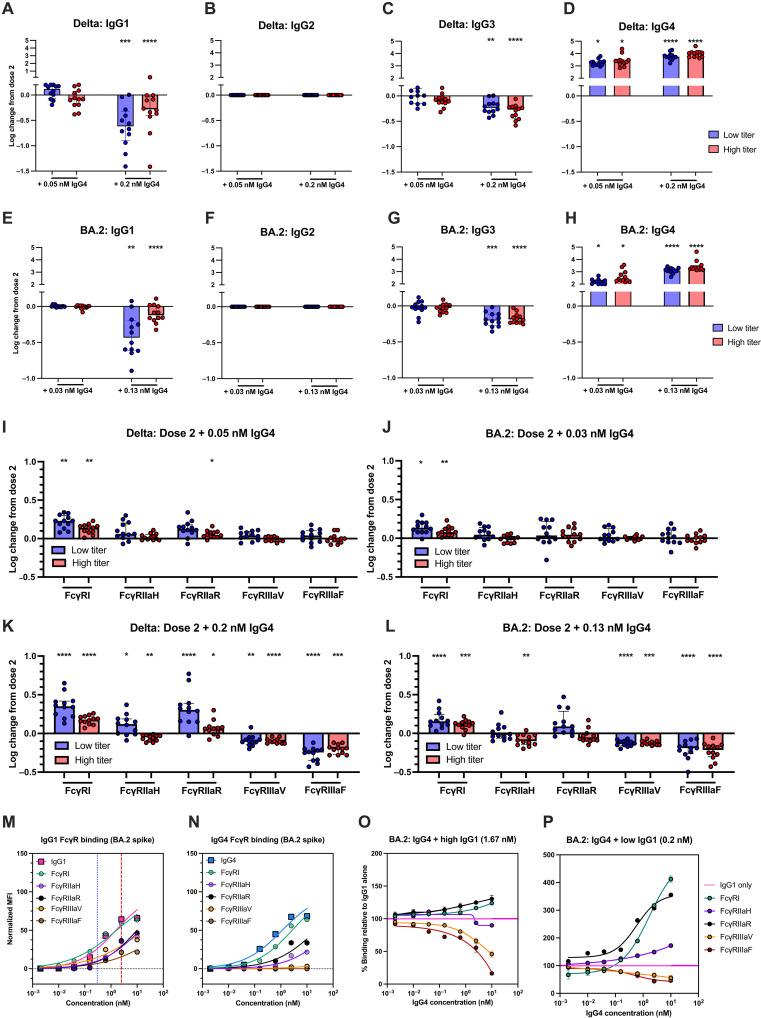
Influence of IgG4 on FcγR mediated responses against SARS-CoV-2 variants. (**A** to **D**) Comparison of [(A) to (D)] Delta and (**E** to **H**) BA.2 spike-bound IgG1 to IgG4 titers post-dose two BNT162b2 vaccination and following IgG4 addition. (**I** to **L**) Change in binding to soluble FcγRs of post-dose two Delta-specific or BA.2-specific antibodies following IgG4 mAb cocktail addition. Significant differences were assessed using Friedman’s test with Dunn’s multiple comparison. Error bars indicate interquartile range. Binding of (**M**) IgG1 and (**N**) IgG4 mAb cocktails to IgG1 or IgG4 detection reagents or recombinant soluble FcγRs against BA.2 Spike trimer. The normalized MFI of each detection reagent was plotted. (**O** to **P**) FcγR binding activity of IgG4 mAb cocktail was titrated into IgG1 mAb cocktail and binding to FcγRs against BA.2 Spike. Error bars indicate SEM. Curves were fitted using a four-parameter nonlinear regression. *P < 0.05, **P < 0.01, ***P < 0.001, and ****P < 0.0001.

As our previous results highlight the importance of total spike-specific antibody titers in determining the influence of increased IgG4 on Fc effector functions and the limited knowledge surrounding the consequence of IgG4 on Fc-mediated responses against SARS-CoV-2 variants, we next investigated the influence of IgG4 on variant-specific Fc-mediated responses As expected, variant-specific IgG1 to IgG4 titers were lower compared to those against ancestral strain. Moreover, we observed a delay in variant-specific subclass switching and weaker negative correlations between variant-specific IgG4 and FcγR binding (fig. S9, A to E). As the plasma samples were collected from COVID-19 naïve individuals who only received ancestral strain mRNA vaccination, we assessed Fc-mediated responses against the Delta and Omicron BA.2 variants as representative novel variants (Fig. 5). We divided our cohort based on their variant spike-specific IgG titers (high/low) and examined spike-specific subclass composition and FcγR binding before and after IgG4 addition. We used the same IgG4 mAbs cocktails, which displays similar binding capacity to Delta in comparsion to the ancestral strain, but reduced binding to BA.2 as one clone does not bind to BA.2, while the other two mAbs display reduced binding to BA.2 (fig. S2 and tables S2 and S3).

Low-concentration IgG4 addition did not significantly change Delta or BA.2 spike binding by IgG1 and IgG3, regardless of spike-specific titers, while high-concentration IgG4 addition significantly reduced Delta and BA.2 spike-binding by IgG1 and IgG3 (Fig. 5, A to H). Consequently at low concentrations, IgG4 addition either enhanced or did not change binding to all tested FcγRs against both variants (Fig. 5, I and J). However, IgG4 addition improved FcγRI binding but reduced FcγRIIIa-158 V/F binding (Fig. 5, K and L). Moreover, Delta-specific FcγRIIa-131H (1.3-fold, *P* < 0.05) and FcγRIIa-131R (2.0-fold, *P* < 0.0001) binding increased in individuals with low Delta spike-specific titers, while individuals with high titers displayed a decrease in FcγRIIaH binding (1.1-fold decrease, *P* < 0.01) and an increase in FcγRIIaR binding (1.1-fold increase, *P* < 0.05). This was further confirmed when replicating conditions with IgG4 mAbs with IgG1 mAb cocktails (fig. S9, H and I).

High-concentration IgG4 addition significantly increased FcγRI binding and decreased FcγRIIIa binding as expected (*P* < 0.001) as expected as well as reduced BA.2-specific FcγRIIaH in individuals with higher BA.2-specific IgG (Fig. 5L). This may be due to the reduced ability of the mAbs to bind BA.2 (ancestral EC_50_: IgG1 = 0.16 nM, IgG4 = 0.19 nM; BA.2 EC_50_: IgG1 = 1.55 nM, IgG4 = 1.14 nM); thus, mAb-mediated FcγR complex formation against BA.2 was also reduced (Fig. 5, M and N). Furthermore, saturation against BA.2 was not observed at the same concentration of IgG1 cocktail where saturation was achieved for ancestral spike (2.5 nM). Consequently, titrating IgG4 into this concentration of IgG1 mirrored results observed when assessing for ancestral responses to IgG4 titration into low levels of IgG1, where we observed improvements in FcγRIIaR and FcγRI binding (Fig. 5O). FcγRIIaH binding was also enhanced when we repeated this with even lower concentrations of IgG1 mAbs (Fig. 5P), emphasizing the functional capacity of IgG4 when total IgG titers are low.

## DISCUSSION

**Fig. 6.**
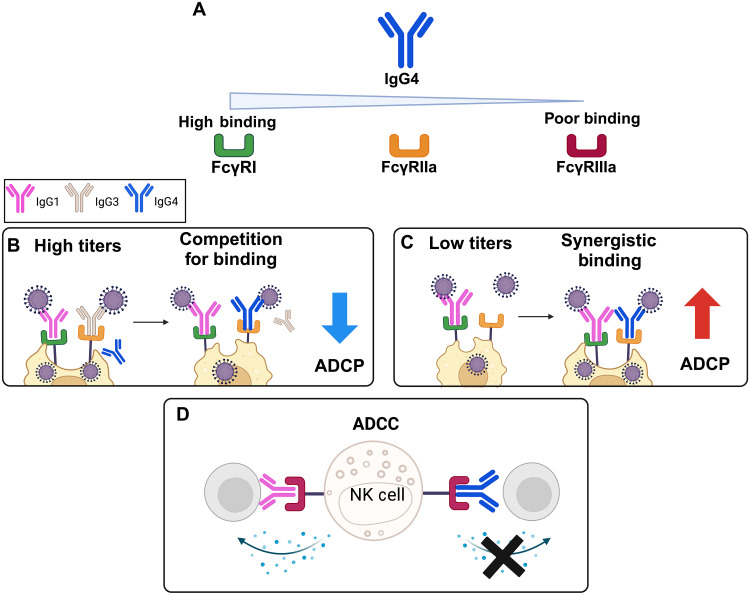
Schematic of the role of anti-spike IgG4 in mediating Fc effector functions against SARS-CoV-2. (**A**) Summary of binding affinity of IgG4 to different FcγRs. (**B**) At high spike-specific IgG titers, elevated IgG4 competes with IgG1 and IgG3 for antigen binding, resulting in reduced FcγRIIa binding and ADCP. (**C**) At low anti-spike titers, increased IgG4 increases total antigen-bound IgG, leading to an overall increase in FcγRI and FcγRIIa binding and therefore ADCP. (**D**) Elevated IgG4 reduces FcγRIIIa engagement and ADCC because of the poor ability of IgG4 to bind FcγRIIIa.

While the rise in IgG4 titers following repeated SARS-CoV-2 mRNA vaccination is well described (*16*–*19*), the clinical and functional impact of this phenomenon is still to be fully elucidated. Here, we demonstrated that elevated spike-specific IgG4 compete with other antibodies for antigen and/or FcγR binding, especially when spike-specific IgG titers are high, resulting in a decrease in Fc effector responses. However, at low spike-specific titers, IgG4 can work in synergy with other subclasses to enhance FcγRI and FcγRIIa-mediated effector functions (summarized in Fig. 6). This suggests elevated spike-specific IgG4 may be advantageous in circumstances where antibody titers are low, such as within immunocompromised individuals (*30*) or in other anatomical sites, such as the mucosa (*16*, *31*). Furthermore, we demonstrate the potential benefit of elevated IgG4 titers for novel variants, where low cross-reactive antibody immunity may be induced by vaccination (*14*, *21*).

Consequently, we demonstrate that elevated IgG4 can impede FcγRIIIa binding and ADCC. In contrast, we show that IgG4 can trigger ADCP by THP-1 monocytes via both FcγRI and FcγRIIa, consistent with a previous study demonstrating this using IgG4 HIV mAbs (*23*). Now, the relative importance of ADCC compared to ADCP in providing protection against COVID-19 in humans is unclear, although some studies have observed elevated ADCC and/or FcγRIIIa binding may be elevated in those with severe disease (*3*, *32*). If excessive ADCC does contribute to severe disease, then elevated IgG4 may be beneficial as it can dampen FcγRIIIa-mediated responses. However, studies in animal models suggest that polyfunctional antibody responses that includes both ADCC and ADCP are correlated with vaccine efficacy (*33*); thus, elevated IgG4 may reduce polyfunctional responses and vaccine induced protection. Note that FcγRIIIa may also be involved in mediating ADCP and conversely FcγRIIa may mediate ADCC (*34*, *35*). Moreover, different effector cells may coexpress different FcγRs at varying proportions (*36*, *37*). Thus, additional studies dissecting the relative contribution of ADCC and ADCP in the context of SARS-CoV-2, while taking account diverse effector cell and their FcγR repertoires is necessary to better understand both functional and clinical consequence of increased IgG4.

Here, we demonstrate that IgG4 was capable of higher levels of binding to FcγRIIaR compared to FcγRIIaH, highlighting the importance of FcγR polymorphisms in determining overall FcγR activation. The distribution of FcγRIIa polymorphisms varies between different ethnicities and geographical locations. FcγRIIaH is more prevalent among East Asian populations than European and African Americans (*38*, *39*); thus, our data suggest that different ethnic populations may benefit more from a rise in IgG4. Now, studies examining the association between FcγR polymorphisms and COVID-19 vaccine efficacy are limited. Nonetheless, FcγRIIaR has been associated with increased disease severity and mortality in both SARS-CoV-2 and SARS-CoV (*40*, *41*), albeit both studies occurring in a small cohort of a single population. Therefore, large genetic studies together with large vaccine efficacy data could be informative.

IgG4 addition into both plasma and IgG1 mAbs demonstrate that IgG4 can compete with other subclasses for antigen binding, and this phenomenon is concentration based. However, multiple factors beyond antibody concentration modulate level of competition between subclasses, including antigen affinity and epitope. Our IgG4 mAb spiking assays likely overestimates the antigen affinity of polyclonal IgG4. It is possible that lower affinity IgG4 may not compete with other subclasses at the concentration used here. However, our IgG4 spiking assays against BA.2, which our mAbs have reduced affinity to antigen, demonstrate that lower affinity IgG4 can outcompete other subclasses at high concentrations. However, we acknowledge our vaccinees induced lower BA.2-specific titers, and future studies should explore whether low-affinity IgG4 outcompetes other subclasses when total titers are high.

Moreover, we do not take into account that different subclasses may have different antigen affinity. IgG4 has been speculated to have the highest antigen affinity among all subclasses as it acquires the most somatic hypermutations among all four subclasses (*42*, *43*), which may allow IgG4 to outcompete other subclasses at lower concentrations. Conversely, IgG4 can undergo F(ab) arm exchange in vivo, whereby IgG4 forms half-molecules, which then recombines with other half-molecules (*20*). Because of the random nature of the exchange, this typically results in a bispecific antibody, which may reduce the avidity of IgG4 (*44*). We did not observe differences in binding kinetics of depleted and undepleted plasma. However, an increase in binding of IgG1 and IgG3 to spike following IgG4 depletion suggests that polyclonal IgG4 has sufficiently high antigen affinity to compete with IgG1 and IgG3 for antigen binding. However, measuring antigen affinity of purified polyclonal IgG4 would be a more accurate measurement and should be performed in the future to improve understanding on the competition between subclasses.

In addition, the mAbs used here target a limited number of epitopes on RBD, which is not a true reflection of polyclonal pool that targets multiple epitopes on spike, including those outside the RBD. Furthermore, our experiments demonstrating competition between IgG1 and IgG4 mAbs may not reflect what occurs in vivo where different subclasses may recognize distinct epitopes. Hence, future studies should aim to investigate IgG4 against epitopes beyond RBD, especially those that are highly conserved across variants and whether these overlap with epitopes recognized by other subclasses. These factors should also be taken into account along with the different subclass affinities to various FcγRs to better understand the importance of antigen binding compared to Fc receptor binding in the induction of Fc-mediated response.

As mentioned previously, the tendency of IgG4 to form bispecific antibodies that behaves as if functionally monovalent is thought to contribute to its reduced capacity to trigger FcγR mediated responses, which often require cross-linking of multiple antigen-specific IgG (*44*). While we did not assess if Fab arm exchange was occurring in our IgG4 mAb cocktail, it is unlikely that immune complex formation is substantially affected should it occur as the target epitopes are in proximity of each other. Future studies should investigate the role of Fab arm exchange on FcγR-mediated responses and the overall consequence of Fc effector functions against SARS-CoV-2.

Overall, our study highlights the complex role of IgG4 in mediating Fc effector functions against SARS-CoV-2. While we have demonstrated that IgG4 exhibits reduced ability to trigger Fc effector functions such as ADCC and ADCP compared to IgG1, the relative contribution of IgG4 in the overall FcγR-mediated response against SARS-CoV-2 was dependent on the titers of each IgG subclass, FcγR class, and polymorphism as well as the target SARS-CoV-2 variant. Collectively, this study emphasizes the interconnected nature of the various factors modulating Fc effector functions. Our work highlights the need for future studies to examine the clinical impact of IgG4 against COVID-19 and to take into consideration the multiple factors that can potentially influence the functional consequences of IgG4.

## MATERIALS AND METHODS

### Human samples and ethics statement

SARS-CoV-2 vaccinee plasma was collected 13 to 42 days post-second (*n* = 46) and post-third (*n* = 31) dose of BNT162b2 (Pfizer-BioNTech) vaccination as previously described (*45*). Participant characteristics are described in table S1. Informed consent was acquired from all participants. Study protocols were approved by the University of Melbourne Human Research Ethics Committee (#2056689, #21560, #21626, and #13344), Austin Health (#HREC/73256/Austin-2021), and Melbourne Health (HREC/68355/MH-2020).

### Expression of SARS-CoV-2-specific mAbs

Spike-specific IgG1 and IgG4 mAbs were expressed as previously described (*21*). PDI 96, PDI 215, and PDI 222 were previously isolated from RBD-reactive B cells from patients with convalescent COVID-19 and cloned into human IgG1 expression vectors (*21*). Class-switched PDI mAb constructs were obtained from GenScript. Expi293F suspension cells were transfected with heavy and light chain plasmids using the ExpiFectamine293 Kit (Life Technologies) according to the manufacturer’s instructions. The cells were harvested after 4 to 5 days, and the antibodies purified by Protein A affinity chromatography.

### BLI of SARS-CoV-2 mAbs

Binding of the mAbs to SARS-CoV-2 RBD and human FcγRs was measured by BLI using an Octet-Red96 system (FortéBio) in black 96-well plates (Greiner Bio-One) at 30°C agitated at 1000 rpm. HBS-EP buffer [containing 0.01 M Hepes, 0.15 M NaCl, 3 mM EDTA, 0.005% (v/v) Surfactant P20, 0.005% Tween 20 (pH 7.4)] was used as the kinetic buffer for all assays.

BLI measuring affinity of the mAbs against human FcγRs were carried out using Streptavidin Biosensors (FortéBio). Biotinylated FcγRIIa or FcγRIIIa (5 μg/ml; ACROBiosystems) were loaded onto hydrated sensors until a threshold of 2 nm was reached, followed by equilibration in buffer only wells for 120 s. The sensors were loaded into a twofold dilution series of mAbs from 27.56 to 441 nM for 180 s to measure association. Dissociation was measured by submerging the sensors into buffer only wells for 300 s. Sensor tips were regenerated using a cycle of 5 s in 10 mM glycine (pH 3.0) and 5 s in kinetic buffer repeated three times. For measurement of affinity to FcγRI, biotinylated mAbs (1.5 μg/ml) were loaded onto hydrated sensors until a threshold of 2 nm was reached, followed by equilibration in buffer only wells for 120 s. The sensors were loaded into a twofold dilution series of FcγRI (R&D Systems) from 2 to 30 nM for 150 s. Dissociation was measured by submerging the sensors into buffer only wells for 300 s. Affinity measurements against SARS-CoV-2 ancestral and variant RBD (SinoBiological) were performed using Protein G Biosensors (FortéBio) and carried out as previously described (*21*).

### IgG4 spiking assays

PDI 96, PDI 215, and PDI 222 were pooled to form an IgG4 mAb cocktail. PDI 222 is part of cluster 1 and is of the VH1–58 class that binds the RBD at an epitope centered around N487. PDI 215 is part of cluster 4 and binds the distal part of the RBD outside the angiotensin-converting enzyme 2 (ACE2) receptor binding site. PDI 96 is part of cluster 6 and binds to the ACE2 binding site at G446 and T500 (*21*). IgG4 mAb cocktail was added into plasma collected post-dose two mRNA vaccination at 0.05 nM (0.007 μg/ml) to represent the median IgG4 concentration measured in our cohort post-third dose mRNA vaccination. Spiking at 0.2 nM (0.029 μg/ml) corresponded to the median IgG4 concentration of a subset of post-dose four mRNA samples (*n* = 6).

### (Fab′)_2_ spiking

Digestion of IgG4 (Fab′)_2_ fragments was performed by incubation of 100 μl of IgG4 mAb cocktail (0.5 mg/ml) with 80 μl of FabRICATOR MagIC resin (Genovis) at 37°C for 20 min with continuous mixing, followed by the removal of the resin by magnetic separation. IgG4 (Fab)_2_ ftragments were added to post-dose two plasma at a concentration of 0.05 nM (0.006 μg/ml) or 0.2 nM (0.022 μg/msl).

### IgG4 depletion

Plasma was diluted 1:5 in phosphate-buffered saline (PBS) before addition of 5 μl of resuspended CaptureSelect IgG4 (Hu) Affinity Matrix (Invitrogen) and incubated on a vortex at room temperature (RT) for 1 hour. Following incubation, the samples were centrifuge for 1 min, and the supernatant was collected. A Luminex multiplex assay was performed to assess efficient IgG4 depletion. Sufficient depletion was defined as >75% reduction in IgG4 relative to the undepleted sample. Depleted samples with IgG4 levels greater than the 25th percentile IgG4 level measured at dose two were excluded from subsequent analysis.

### SARS-CoV-2 multiplex bead assay

A customized Luminex multiplex assay was used to assess mAb and plasma SARS-CoV-2 antibody responses as previously described (*46*). Briefly, mAbs or plasma (1:1600) was incubated with antigen-coupled beads (750 beads/bead region; table S4) on a shaker overnight at 4°C before being washed with PBS 0.05% Tween 20 (PBST). Following washing, the beads were incubated with biotinylated or phycoerythrin (PE)–conjugated detection antibodies or soluble Fc receptors (table S5) on a shaker for 2 hours at RT. For biotinylated detectors, plates were followed with an additional PBST wash and incubated with streptavidin-R–PE (Invitrogen) for 2 hours at RT. The plates were washed, and each well was resuspended sheath fluid before reading on the xMAP INTELLIFLEX (Luminex). The level of PE signal associated with each bead region in each well was reported as median fluorescence intensity (MFI). The MFI of buffer only wells were background subtracted for each sample. Standard curves were generated using IgG1 to IgG4 anti–SARS-CoV-2 RBD broadly neutralizing mAbs (AM395b, Acro Biosystems). A four-parameter logistic regression was used to convert the MFI of plasma samples to antigen-specific IgG1 to IgG4 concentrations, relative to the standard curve. Individuals with total relative antigen-specific IgG titers below the median were classified as low titer, and individuals with total titers above the median were classified as high titer. Each sample was run in duplicate.

### Bead-based THP-1 ADCP assay

ADCP was measured using a previously described bead-based ADCP assay (*24*, *25*). Briefly, SARS-CoV-2 ancestral spike trimer and RBD (SinoBiological) were biotinylated and coupled to 1 μM fluorescent NeutrAvidin Fluospheres (beads; Invitrogen) overnight at 4°C. Washed antigen-coated beads were incubated with plasma (1:3200) or mAbs for 2 hours at 37°C in a 96-well U-bottom plate before addition of THP-1 monocytes (100,000 per well) followed by a 16-hour incubation under cell culture conditions. For Fc block experiments, THP-1 monocytes were incubated BD Pharmingen Human BD Fc Block at RT for 20 min (2.5 μg/1 million cells) before addition to opsonized beads. Cells were fixed and acquired by flow cytometry on a BD LSR Fortessa with a high-throughput sampler attachment (HTS) (see fig. S3A for gating). The data were analyzed using FlowJo 10.9.0, and a phagocytic score was calculated as previously described using the formula: (% bead-positive cells × mean fluorescent intensity). Flow cytometry was used to confirm stable expression of FcγRI (CD64), FcγRII (CD32), and FcαR (CD89) (table S4) on THP-1 monocytes before use.

### Luciferase-based ADCC assay

ADCC was examined using a previously described luciferase-based ADCC assay (*24*). NK cells from healthy donors were enriched and purified using the EasySep Human NK Cell Enrichment Kit (STEMCELL Technologies Inc.) NK cells (20,000 per well) and Ramos S-Luc cells (5000 per well) were added to 96-well V-bottom cell culture plates and incubated with threefold dilutions of mAb cocktail for 4 hours at 37°C. Each condition was tested in duplicate, and “no antibody” and “target cell only” controls were included. Following incubation, cells were washed and developed with britelite plus luciferase reagent (Revvity). Luminescence was read using a FLUOstar Omega microplate reader (BMG Labtech). The relative light units measured was used to calculate % ADCC with the formula: (“no antibody” − “antibody sample”)/(“target cell only”) × 100.

### BLI measurements of IgG4-depleted and undepleted plasma

To measure binding kinetics of IgG4-depleted plasma and undepleted plasma to spike, hydrated streptavidin sensors were loaded with biotinylated ancestral SARS-CoV-2 spike trimer (5 μg/ml) until a threshold of 1.6 nm was reached, followed by equilibration in buffer only wells for 60 s. The sensors were submerged in a twofold dilution series of plasma or IgG4-depleted plasma, starting from a 1 in 400 dilution, for 180 s to allow for association. Dissociation was measured by submerging sensors into buffer only wells for 300 s. Only the dissociation rate constant (kdis), which is a concentration-independent measurement was measured, given that plasma consists of polyclonal antibodies (*28*, *29*). Curve fitting was performed using a local fit 1:1 Langmuir binding model for plasma. Baseline drift was corrected by reference subtracting the shift of a loaded sensor immersed in kinetic buffer only. Mean kinetic constant and SEM were determined from two independent experiments.

### Data normalization

The antibody MFI and phagocytic score of each mAb cocktail was normalized to the maximum antibody binding or phagocytic score (100%). The normalized antibody binding or phagocytic score was used to calculate the half maximal effective concentration (EC_50_). For IgG4 mAb cocktail titration into IgG1 mAb cocktail experiments, the antibody MFI, phagocytic score, or % ADCC of IgG1 + IgG4 mAb cocktails were normalized to the antibody binding, phagocytic score, or % ADCC of the IgG1 mAb cocktail alone.

### In silico FcγR ODE model generation and validation

FcγRIIa binding simulations were carried out using a previously published ODE model (*26*) implemented in MATLAB (R2023b, MathWorks) that predicts FcγR complex formation as a function of IgG subclass concentrations. Model parameters are outlined in table S6. The model was used to predict FcγRIIa-131H and FcγRIIa-131R complex formation as a function of measured IgG subclass concentrations for each dose 2 mRNA-vaccinated plasma samples. To fit the model to the data, measured IgG concentrations were proportionally adjusted by a constant factor (λ = 25) to maximize the fit with the corresponding predictions from the corresponding multiplex assay measurements. Code and data used for these models are available at https://doi.org/10.5281/zenodo.14231188.

### Surface plots to evaluate the combined effects of IgG1 and IgG4

FcγR complex formation surfaces were generated by simulating the model across 50 IgG1 concentrations and 100 IgG4 concentrations logarithmically spaced from 0.10 to 10 nM and 0.045 to 7.5 nM respectively to cover the plasma concentration range observed before and after the addition of IgG4 mAb. IgG2 and IgG3 levels were set to the median values in the dose two mRNA-vaccinated samples. Surface color was determined by numerical approximation of the partial derivative of FcγR complex formation with respect to IgG4 concentration at each IgG1 and IgG4 combination (gradient function).

### Statistical analysis

Prism GraphPad version 10.1.0 (GraphPad Software) was used to develop graphs and perform the statistical analyses described in the figure legends. The EC_50_ of each mAb or mAb cocktail was determined using a four-parameter nonlinear regression model describing the relationship between the agonist concentration and the normalized response.

## References

[1] D. S. Khoury, D. Cromer, A. Reynaldi, T. E. Schlub, A. K. Wheatley, J. A. Juno, K. Subbarao, S. J. Kent, J. A. Triccas, M. P. Davenport, Neutralizing antibody levels are highly predictive of immune protection from symptomatic SARS-CoV-2 infection. Nat. Med. 27, 1205–1211 (2021). 34002089 10.1038/s41591-021-01377-8

[2] T. Zohar, C. Loos, S. Fischinger, C. Atyeo, C. Wang, M. D. Slein, J. Burke, J. Yu, J. Feldman, B. M. Hauser, T. Caradonna, A. G. Schmidt, Y. Cai, H. Streeck, E. T. Ryan, D. H. Barouch, R. C. Charles, D. A. Lauffenburger, G. Alter, Compromised humoral functional evolution tracks with SARS-CoV-2 mortality. Cell 183, 1508–1519.e12 (2020). 33207184 10.1016/j.cell.2020.10.052 PMC7608014

[3] C. Atyeo, S. Fischinger, T. Zohar, M. D. Slein, J. Burke, C. Loos, D. J. McCulloch, K. L. Newman, C. Wolf, J. Yu, K. Shuey, J. Feldman, B. M. Hauser, T. Caradonna, A. G. Schmidt, T. J. Suscovich, C. Linde, Y. Cai, D. Barouch, E. T. Ryan, R. C. Charles, D. Lauffenburger, H. Chu, G. Alter, Distinct early serological signatures track with SARS-CoV-2 survival. Immunity 53, 524–532.e4 (2020). 32783920 10.1016/j.immuni.2020.07.020 PMC7392190

[4] A. Schäfer, F. Muecksch, J. C. C. Lorenzi, S. R. Leist, M. Cipolla, S. Bournazos, F. Schmidt, R. M. Maison, A. Gazumyan, D. R. Martinez, R. S. Baric, D. F. Robbiani, T. Hatziioannou, J. V. Ravetch, P. D. Bieniasz, R. A. Bowen, M. C. Nussenzweig, T. P. Sheahan, Antibody potency, effector function, and combinations in protection and therapy for SARS-CoV-2 infection in vivo. J. Exp. Med. 218, e20201993 (2021). 33211088 10.1084/jem.20201993 PMC7673958

[5] K. B. Arnold, A. W. Chung, Prospects from systems serology research. Immunology 153, 279–289 (2018). 29139548 10.1111/imm.12861 PMC5795183

[6] S. R. Mackin, P. Desai, B. M. Whitener, C. E. Karl, M. Liu, R. S. Baric, D. K. Edwards, T. M. Chicz, R. P. McNamara, G. Alter, M. S. Diamond, Fc-γR-dependent antibody effector functions are required for vaccine-mediated protection against antigen-shifted variants of SARS-CoV-2. Nat. Microbiol. 8, 569–580 (2023). 37012355 10.1038/s41564-023-01359-1 PMC10797606

[7] E. R. Haycroft, S. K. Davis, P. Ramanathan, E. Lopez, R. A. Purcell, L. L. Tan, P. Pymm, B. D. Wines, P. M. Hogarth, A. K. Wheatley, J. A. Juno, S. J. Redmond, N. A. Gherardin, D. I. Godfrey, W. H. Tham, K. J. Selva, S. J. Kent, A. W. Chung, Antibody Fc-binding profiles and ACE2 affinity to SARS-CoV-2 RBD variants. Med. Microbiol. Immunol. 212, 291–305 (2023). 37477828 10.1007/s00430-023-00773-w PMC10372118

[8] P. Kaplonek, S. Fischinger, D. Cizmeci, Y. C. Bartsch, J. Kang, J. S. Burke, S. A. Shin, D. Dayal, P. Martin, C. Mann, F. Amanat, B. Julg, E. J. Nilles, E. R. Musk, A. S. Menon, F. Krammer, E. O. Saphire, C. Andrea, G. Alter, mRNA-1273 vaccine-induced antibodies maintain Fc effector functions across SARS-CoV-2 variants of concern. Immunity 55, 355–365.e4 (2022). 35090580 10.1016/j.immuni.2022.01.001 PMC8733218

[9] S. I. Richardson, N. P. Manamela, B. M. Motsoeneng, H. Kaldine, F. Ayres, Z. Makhado, M. Mennen, S. Skelem, N. Williams, N. J. Sullivan, J. Misasi, G. G. Gray, L.-G. Bekker, V. Ueckermann, T. M. Rossouw, M. T. Boswell, N. A. B. Ntusi, W. A. Burgers, P. L. Moore, SARS-CoV-2 Beta and Delta variants trigger Fc effector function with increased cross-reactivity. Cell Rep. Med. 3, 100510 (2022). 35233544 10.1016/j.xcrm.2022.100510 PMC8761540

[10] G. Vidarsson, G. Dekkers, T. Rispens, IgG Subclasses and allotypes: From structure to effector functions. Front. Immunol. 5, 520 (2014). 25368619 10.3389/fimmu.2014.00520 PMC4202688

[11] P. Bruhns, B. Iannascoli, P. England, D. A. Mancardi, N. Fernandez, S. Jorieux, M. Daëron, Specificity and affinity of human Fcγ receptors and their polymorphic variants for human IgG subclasses. Blood 113, 3716–3725 (2009). 19018092 10.1182/blood-2008-09-179754

[12] A. W. Chung, M. Ghebremichael, H. Robinson, E. Brown, I. Choi, S. Lane, A.-S. Dugast, M. K. Schoen, M. Rolland, T. J. Suscovich, A. E. Mahan, L. Liao, H. Streeck, C. Andrews, S. Rerks-Ngarm, S. Nitayaphan, M. S. de Souza, J. Kaewkungwal, P. Pitisuttithum, D. Francis, N. L. Michael, J. H. Kim, C. Bailey-Kellogg, M. E. Ackerman, G. Alter, Polyfunctional Fc-effector profiles mediated by IgG subclass selection distinguish RV144 and VAX003 vaccines. Sci. Transl. Med. 6, 228ra38 (2014). 10.1126/scitranslmed.3007736 24648341

[13] T. Damelang, S. J. Rogerson, S. J. Kent, A. W. Chung, Role of IgG3 in infectious diseases. Trends Immunol. 40, 197–211 (2019). 30745265 10.1016/j.it.2019.01.005

[14] D. N. Forthal, G. Landucci, H. Ding, J. C. Kappes, A. Wang, I. Thung, T. Phan, IgG2 inhibits HIV-1 internalization by monocytes, and IgG subclass binding is affected by gp120 glycosylation. AIDS 25, 2099–2104 (2011). 21832933 10.1097/QAD.0b013e32834b64bd

[15] P. Karagiannis, A. E. Gilbert, D. H. Josephs, N. Ali, T. Dodev, L. Saul, I. Correa, L. Roberts, E. Beddowes, A. Koers, C. Hobbs, S. Ferreira, J. L. C. Geh, C. Healy, M. Harries, K. M. Acland, P. J. Blower, T. Mitchell, D. J. Fear, J. F. Spicer, K. E. Lacy, F. O. Nestle, S. N. Karagiannis, IgG4 subclass antibodies impair antitumor immunity in melanoma. J. Clin. Invest. 123, 1457–1474 (2013). 23454746 10.1172/JCI65579 PMC3613918

[16] K. J. Selva, P. Ramanathan, E. R. Haycroft, A. Reynaldi, D. Cromer, C. W. Tan, L. F. Wang, B. D. Wines, P. M. Hogarth, L. E. Downie, S. K. Davis, R. A. Purcell, H. E. Kent, J. A. Juno, A. K. Wheatley, M. P. Davenport, S. J. Kent, A. W. Chung, Preexisting immunity restricts mucosal antibody recognition of SARS-CoV-2 and Fc profiles during breakthrough infections. JCI Insight 8, e172470 (2023). 37737263 10.1172/jci.insight.172470 PMC10561726

[17] P. Irrgang, J. Gerling, K. Kocher, D. Lapuente, P. Steininger, K. Habenicht, M. Wytopil, S. Beileke, S. Schäfer, J. Zhong, G. Ssebyatika, T. Krey, V. Falcone, C. Schülein, A. S. Peter, K. Nganou-Makamdop, H. Hengel, J. Held, C. Bogdan, K. Überla, K. Schober, T. H. Winkler, M. Tenbusch, Class switch toward noninflammatory, spike-specific IgG4 antibodies after repeated SARS-CoV-2 mRNA vaccination. Sci. Immunol. 8, (2023). 10.1126/sciimmunol.ade2798 PMC9847566 36548397

[18] N. Lasrado, A.-R. Y. Collier, J. Miller, N. P. Hachmann, J. Liu, T. Anand, E. A. Bondzie, J. L. Fisher, C. R. Mazurek, R. C. Patio, S. L. Rodrigues, M. Rowe, N. Surve, D. M. Ty, C. Wu, T. M. Chicz, X. Tong, B. Korber, R. P. Mcnamara, D. H. Barouch, Waning immunity and IgG4 responses following bivalent mRNA boosting. Sci. Adv. 8, eadj9945 (2024). 10.1126/sciadv.adj9945 PMC10889350 38394195

[19] R. Kalkeri, M. Zhu, S. Cloney-Clark, J. S. Plested, A. Parekh, D. Gorinson, R. Cai, S. Mahato, P. Ramanathan, L. C. Aurelia, K. J. Selva, A. M. Marchese, L. Fries, A. W. Chung, L. M. Dunkle, Altered IgG4 antibody response to repeated mRNA versus recombinant protein SARS-CoV-2 vaccines. J. Infect. 88, 106119 (2024). 38360356 10.1016/j.jinf.2024.106119

[20] T. Rispens, M. G. Huijbers, The unique properties of IgG4 and its roles in health and disease. Nat. Rev. Immunol. 23, 763–778 (2023). 37095254 10.1038/s41577-023-00871-z PMC10123589

[21] A. K. Wheatley, P. Pymm, R. Esterbauer, M. H. Dietrich, W. S. Lee, D. Drew, H. G. Kelly, L.-J. Chan, F. L. Mordant, K. A. Black, A. Adair, H.-X. Tan, J. A. Juno, K. M. Wragg, T. Amarasena, E. Lopez, K. J. Selva, E. R. Haycroft, J. P. Cooney, H. Venugopal, L. L. Tan, M. T. O. Neill, C. C. Allison, D. Cromer, M. P. Davenport, R. A. Bowen, A. W. Chung, M. Pellegrini, M. T. Liddament, A. Glukhova, K. Subbarao, S. J. Kent, W.-H. Tham, Landscape of human antibody recognition of the SARS-CoV-2 receptor binding domain. Cell Rep. 37, 109822 (2021). 34610292 10.1016/j.celrep.2021.109822 PMC8463300

[22] M. Brüggemann, G. T. Williams, C. I. Bindon, M. R. Clark, M. R. Walker, R. Jefferis, H. Waldmann, M. S. Neuberger, Comparison of the effector functions of human immunoglobulins using a matched set of chimeric antibodies. J. Exp. Med. 166, 1351–1361 (1987). 3500259 10.1084/jem.166.5.1351 PMC2189658

[23] J. M. Brady, M. Phelps, S. W. MacDonald, E. C. Lam, A. Nitido, D. Parsons, C. L. Boutros, C. E. Deal, W. F. Garcia-Beltran, S. Tanno, H. Natarajan, M. E. Ackerman, V. D. Vrbanac, A. B. Balazs, Antibody-mediated prevention of vaginal HIV transmission is dictated by IgG subclass in humanized mice. Sci. Transl. Med. 14, eabn9662 (2022). 35895834 10.1126/scitranslmed.abn9662 PMC9507259

[24] W. S. Lee, K. J. Selva, S. K. Davis, B. D. Wines, A. Reynaldi, R. Esterbauer, H. G. Kelly, E. R. Haycroft, H. X. Tan, J. A. Juno, A. K. Wheatley, P. M. Hogarth, D. Cromer, M. P. Davenport, A. W. Chung, S. J. Kent, Decay of Fc-dependent antibody functions after mild to moderate COVID-19. Cell Rep. Med. 2, 100296 (2021). 33997824 10.1016/j.xcrm.2021.100296 PMC8106889

[25] S. K. Davis, K. J. Selva, E. Lopez, E. R. Haycroft, W. S. Lee, A. K. Wheatley, J. A. Juno, A. Adair, P. Pymm, S. J. Redmond, N. A. Gherardin, D. I. Godfrey, W. Tham, S. J. Kent, A. W. Chung, Heterologous SARS-CoV-2 IgA neutralising antibody responses in convalescent plasma. Clin. Transl. Immunology 11, e1424 (2022). 36299410 10.1002/cti2.1424 PMC9588388

[26] M. M. Lemke, M. R. McLean, C. Y. Lee, E. Lopez, E. R. Bozich, S. Rerks-Ngarm, P. Pitisuttithum, S. Nitayaphan, S. Kratochvil, B. D. Wines, P. M. Hogarth, S. J. Kent, A. W. Chung, K. B. Arnold, A systems approach to elucidate personalized mechanistic complexities of antibody-Fc receptor activation post-vaccination. Cell Rep. Med. 2, 100386 (2021). 34622227 10.1016/j.xcrm.2021.100386 PMC8484512

[27] M. M. Lemke, R. M. Theisen, E. R. Bozich, M. R. McLean, C. Y. Lee, E. Lopez, S. Rerks-Ngarm, P. Pitisuttithum, S. Nitayaphan, S. Kratochvil, B. D. Wines, P. M. Hogarth, S. J. Kent, A. W. Chung, K. B. Arnold, A Quantitative approach to unravel the role of host genetics in IgG-FcγR complex formation after vaccination. Front. Immunol. 13, 820148 (2022). 35273603 10.3389/fimmu.2022.820148 PMC8902241

[28] P. J. Klasse, How to assess the binding strength of antibodies elicited by vaccination against HIV and other viruses. Expert Rev. Vaccines 15, 295–311 (2016). 26641943 10.1586/14760584.2016.1128831 PMC4766047

[29] V. Madhavi, B. D. Wines, J. Amin, S. Emery, ENCORE Study Group, E. Lopez, A. Kelleher, Sydney LTNP Study Group, R. J. Center, P. M. Hogarth, A. W. Chung, S. J. Kent, I. Stratov, HIV-1 Env- and Vpu-specific antibody-dependent cellular cytotoxicity responses associated with elite control of HIV. J. Virol. 91, e00700-17 (2017). 28701393 10.1128/JVI.00700-17 PMC5571238

[30] R. A. Purcell, R. M. Theisen, K. B. Arnold, A. W. Chung, K. J. Selva, Polyfunctional antibodies: A path towards precision vaccines for vulnerable populations. Front. Immunol. 14, 1183727 (2023). 37600816 10.3389/fimmu.2023.1183727 PMC10433199

[31] K. J. Selva, S. K. Davis, E. R. Haycroft, W. S. Lee, E. Lopez, A. Reynaldi, M. P. Davenport, H. E. Kent, J. A. Juno, A. W. Chung, S. J. Kent, Tear antibodies to SARS-CoV-2: Implications for transmission. Clin. Transl. Immunology 10, e1354 (2021). 34754451 10.1002/cti2.1354 PMC8559894

[32] S. E. Butler, A. R. Crowley, H. Natarajan, S. Xu, J. A. Weiner, C. A. Bobak, D. E. Mattox, J. Lee, W. Wieland-Alter, R. I. Connor, P. F. Wright, M. E. Ackerman, Distinct features and functions of systemic and mucosal humoral immunity among SARS-CoV-2 convalescent individuals. Front. Immunol. 11, 618685 (2021). 33584712 10.3389/fimmu.2020.618685 PMC7876222

[33] K. McMahan, J. Yu, N. B. Mercado, C. Loos, L. H. Tostanoski, A. Chandrashekar, J. Liu, L. Peter, C. Atyeo, A. Zhu, E. A. Bondzie, G. Dagotto, M. S. Gebre, C. Jacob-Dolan, Z. Li, F. Nampanya, S. Patel, L. Pessaint, A. Van Ry, K. Blade, J. Yalley-Ogunro, M. Cabus, R. Brown, A. Cook, E. Teow, H. Andersen, M. G. Lewis, D. A. Lauffenburger, G. Alter, D. H. Barouch, Correlates of protection against SARS-CoV-2 in rhesus macaques. Nature 590, 630–634 (2021). 33276369 10.1038/s41586-020-03041-6 PMC7906955

[34] R. F. Graziano, Fc gamma RI and Fc gamma RII on monocytes and granulocytes are cytotoxic trigger molecules for tumor cells. J. Immunol. 139, 3536–3541 (1987). 2960735

[35] C. L. Anderson, L. Shen, D. M. Eicher, M. D. Wewers, J. K. Gill, Phagocytosis mediated by three distinct Fc gamma receptor classes on human leukocytes. J. Exp. Med. 171, 1333–1345 (1990). 2139103 10.1084/jem.171.4.1333 PMC2187839

[36] C. L. Anderson, R. J. Loone, Human leukocyte IgG Fc receptors. Immunol. Today 7, 264–266 (1986). 25290629 10.1016/0167-5699(86)90007-1

[37] J. C. Unkeless, Human Fc receptors for IgG. Int. Rev. Immunol. 5, 165–171 (1989). 8691049 10.3109/08830188909061983

[38] T. Lehrnbecher, C. B. Foster, S. Zhu, S. F. Leitman, L. R. Goldin, K. Huppi, S. J. Chanock, Variant genotypes of the low-affinity Fc receptors in two control populations and a review of low-affinity Fc receptor polymorphisms in control and disease populations. Blood 94, 4220–4232 (1999). 10590067

[39] J. M. Osborne, G. W. Chacko, J. T. Brandt, C. L. Anderson, Ethnic variation in frequency of an allelic polymorphism of human FcyRIIA determined with allele specific oligonucleotide probes. J. Immunol. Methods 173, 207–217 (1994). 8046255 10.1016/0022-1759(94)90299-2

[40] R. López-Martínez, G. M. Albaiceta, L. Amado-Rodríguez, E. Cuesta-Llavona, J. Gómez, M. García-Clemente, D. Vázquez-Coto, V. Alvarez, E. Coto, The FCGR2A rs1801274 polymorphism was associated with the risk of death among COVID-19 patients. Clin. Immunol. 236, 108954 (2022). 35149195 10.1016/j.clim.2022.108954 PMC8824710

[41] F. F. Yuan, J. Tanner, P. K. S. Chan, S. Biffin, W. B. Dyer, A. F. Geczy, J. W. Tang, D. S. C. Hui, J. J. Y. Sung, J. S. Sullivan, Influence of *FcγRIIA* and *MBL* polymorphisms on severe acute respiratory syndrome. Tissue Antigens 66, 291–296 (2005). 16185324 10.1111/j.1399-0039.2005.00476.x PMC7190181

[42] K. Kitaura, H. Yamashita, H. Ayabe, T. Shini, T. Matsutani, R. Suzuki, Different somatic hypermutation levels among antibody subclasses disclosed by a new next-generation sequencing-based antibody repertoire analysis. Front. Immunol. 8, 389 (2017). 28515723 10.3389/fimmu.2017.00389 PMC5413556

[43] K. J. L. Jackson, Y. Wang, A. M. Collins, Human immunoglobulin classes and subclasses show variability in VDJ gene mutation levels. Immunol. Cell Biol. 92, 729–733 (2014). 24913324 10.1038/icb.2014.44

[44] M. van der Neut Kolfschoten, J. Schuurman, M. Losen, W. K. Bleeker, P. Martínez-Martínez, E. Vermeulen, T. H. den Bleker, L. Wiegman, T. Vink, L. A. Aarden, M. H. De Baets, J. G. J. van de Winkel, R. C. Aalberse, P. W. H. I. Parren, Anti-inflammatory activity of human IgG4 antibodies by dynamic Fab arm exchange. Science 317, 1554–1557 (2007). 17872445 10.1126/science.1144603

[45] R. A. Purcell, L. C. Aurelia, R. Esterbauer, L. F. Allen, K. A. Bond, D. A. Williamson, J. M. Trevillyan, J. A. Trubiano, J. J. Juno, A. K. Wheatley, M. P. Davenport, T. H. O. Nguyen, K. Kedzierska, S. J. Kent, K. J. Selva, A. W. Chung, Immunoglobulin G genetic variation can confound assessment of antibody levels via altered binding to detection reagents. Clin. Transl. Immunology 13, e1494 (2024). 38433763 10.1002/cti2.1494 PMC10902689

[46] K. J. Selva, C. E. van de Sandt, M. M. Lemke, C. Y. Lee, S. K. Shoffner, B. Y. Chua, S. K. Davis, T. H. O. Nguyen, L. C. Rowntree, L. Hensen, M. Koutsakos, C. Y. Wong, F. Mordant, D. C. Jackson, K. L. Flanagan, J. Crowe, S. Tosif, M. R. Neeland, P. Sutton, P. V. Licciardi, N. W. Crawford, A. C. Cheng, D. L. Doolan, F. Amanat, F. Krammer, K. Chappell, N. Modhiran, D. Watterson, P. Young, W. S. Lee, B. D. Wines, P. M. Hogarth, R. Esterbauer, H. G. Kelly, H.-X. Tan, J. A. Juno, A. K. Wheatley, S. J. Kent, K. B. Arnold, K. Kedzierska, A. W. Chung, Systems serology detects functionally distinct coronavirus antibody features in children and elderly. Nat. Commun. 12, 2037 (2021). 33795692 10.1038/s41467-021-22236-7 PMC8016934

