# Supplementary material for: Increased SARS-CoV-2 IgG4 has variable consequences dependent upon Fc function, Fc receptor polymorphism, and viral variant
Source: Sci Adv. 2025 Feb 26;11(9):eads1482. doi: 10.1126/sciadv.ads1482 (PMC11864192; doi:10.1126/sciadv.ads1482)
Supplement: Supplementary file 1 — Figs. S1 to S9 Tables S1 to S6 [file sciadv.ads1482_sm.pdf]

Science Advances
AAAS

Supplementary Materials for

Increased SARS-CoV-2 IgG4 has variable consequences dependent upon Fc function, Fc receptor polymorphism, and viral variant

L. Carissa Aurelia *et al.*

Corresponding author: Amy W. Chung, awchung@unimelb.edu.au

Sci. Adv. **11**, eads1482 (2025)
DOI: 10.1126/sciadv.ads1482

This PDF file includes:

Figs. S1 to S9
Tables S1 to S6

SUPPLEMENTARY FIGURES

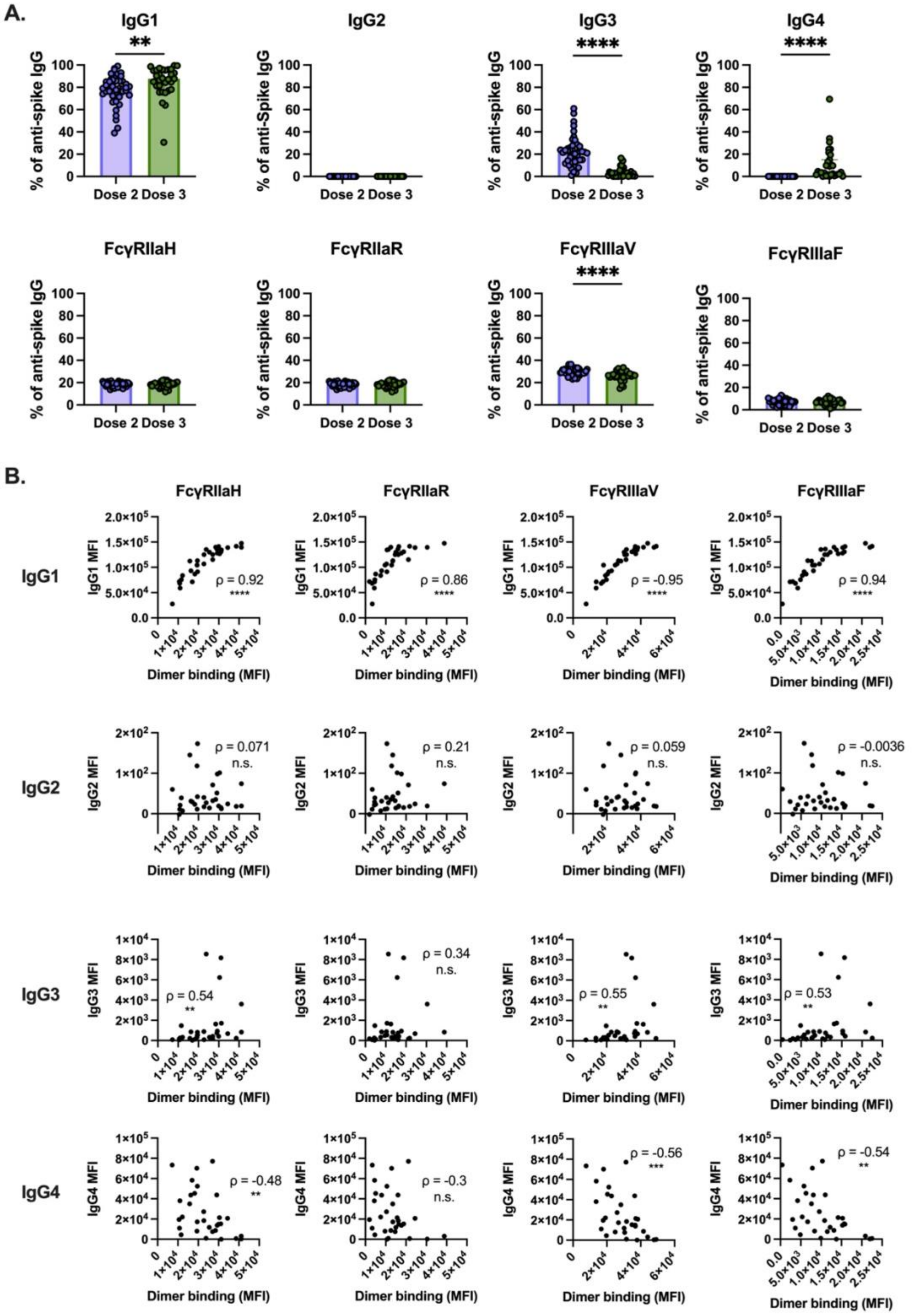

Fig S1. Ancestral spike-specific IgG responses following repeated mRNA vaccination. (A) Plasma IgG1-4 and FcγR binding activity against ancestral Spike trimer following two and three doses of BNT162b2 vaccination. Error bars indicate interquartile range. Statistical

significance was assessed using Mann-Whitney *U*-test. **(B)** Spearman correlation between ancestral spike trimer-specific IgG1-4 titres and binding of ancestral spike trimer-specific FcγR binding. n.s. not significant, $**p<0.01$, $***p<0.001$, $****p<0.0001$.

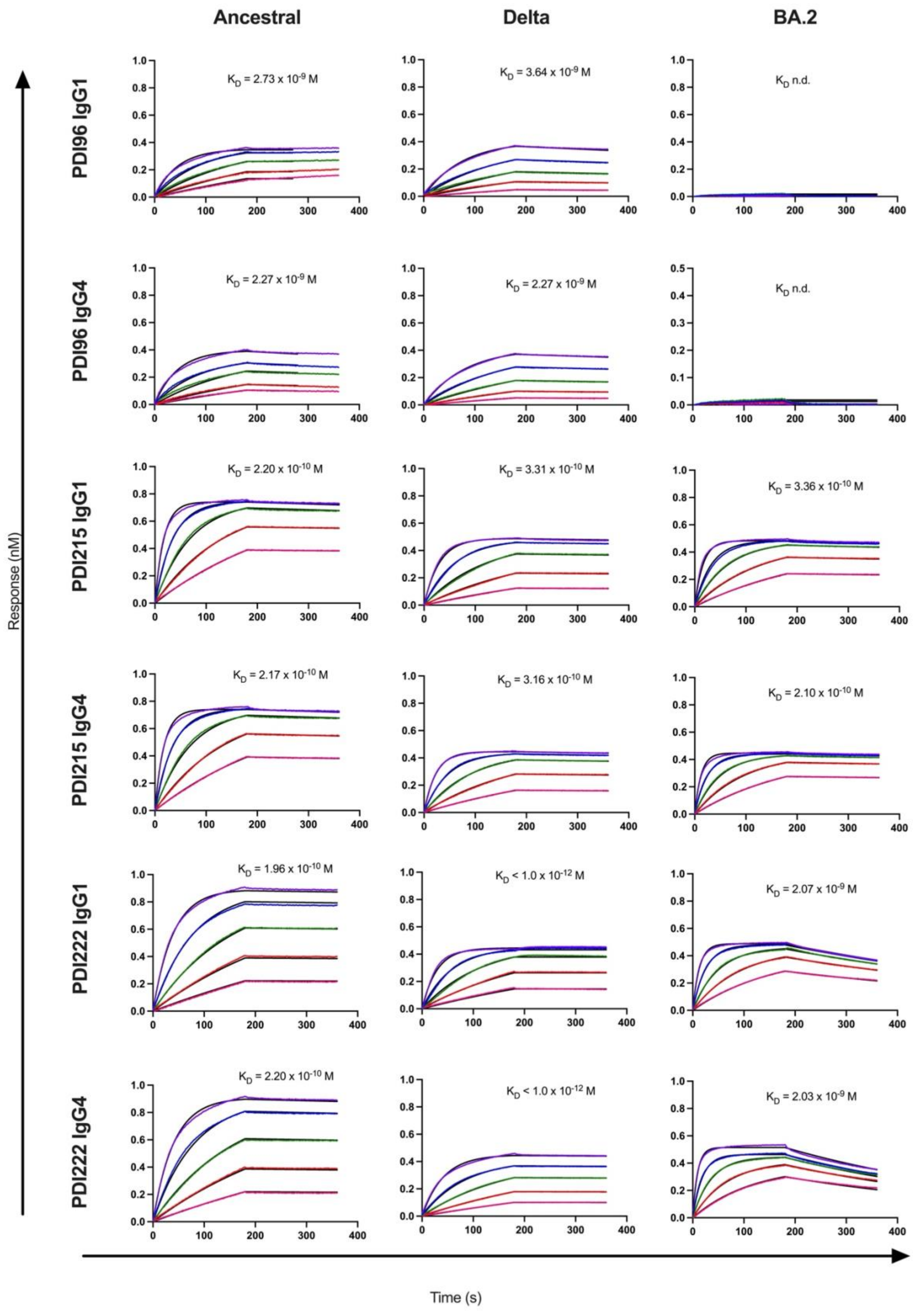

Ancestral
Delta
BA.2
PDI96 IgG1
PDI96 IgG4
PDI215 IgG1
PDI215 IgG4
PDI222 IgG1
PDI222 IgG4
K_D = 2.73 x 10^-9 M
K_D = 3.64 x 10^-9 M
K_D n.d.
K_D = 2.27 x 10^-9 M
K_D = 2.27 x 10^-9 M
K_D n.d.
K_D = 2.20 x 10^-10 M
K_D = 3.31 x 10^-10 M
K_D = 3.36 x 10^-10 M
K_D = 2.17 x 10^-10 M
K_D = 3.16 x 10^-10 M
K_D = 2.10 x 10^-10 M
K_D = 1.96 x 10^-10 M
K_D < 1.0 x 10^-12 M
K_D = 2.07 x 10^-9 M
K_D = 2.20 x 10^-10 M
K_D < 1.0 x 10^-12 M
K_D = 2.03 x 10^-9 M
Response (nM)
Time (s)

Fig S2. Binding of PDI monoclonal antibodies to SARS-CoV-2 receptor binding domain. Bio-layer interferometry sensograms of interaction immobilised IgG1 or IgG4 SARS-CoV-2 monoclonal antibodies with SARS-CoV-2 receptor binding domain (RBD) from the ancestral, Delta and Omicron BA.2 strains. The RBD was diluted serially two-folds. The curves were fitted based on the 1:1 binding kinetic model using data analysis software 12.0 (FortéBio). n.d: not determined.

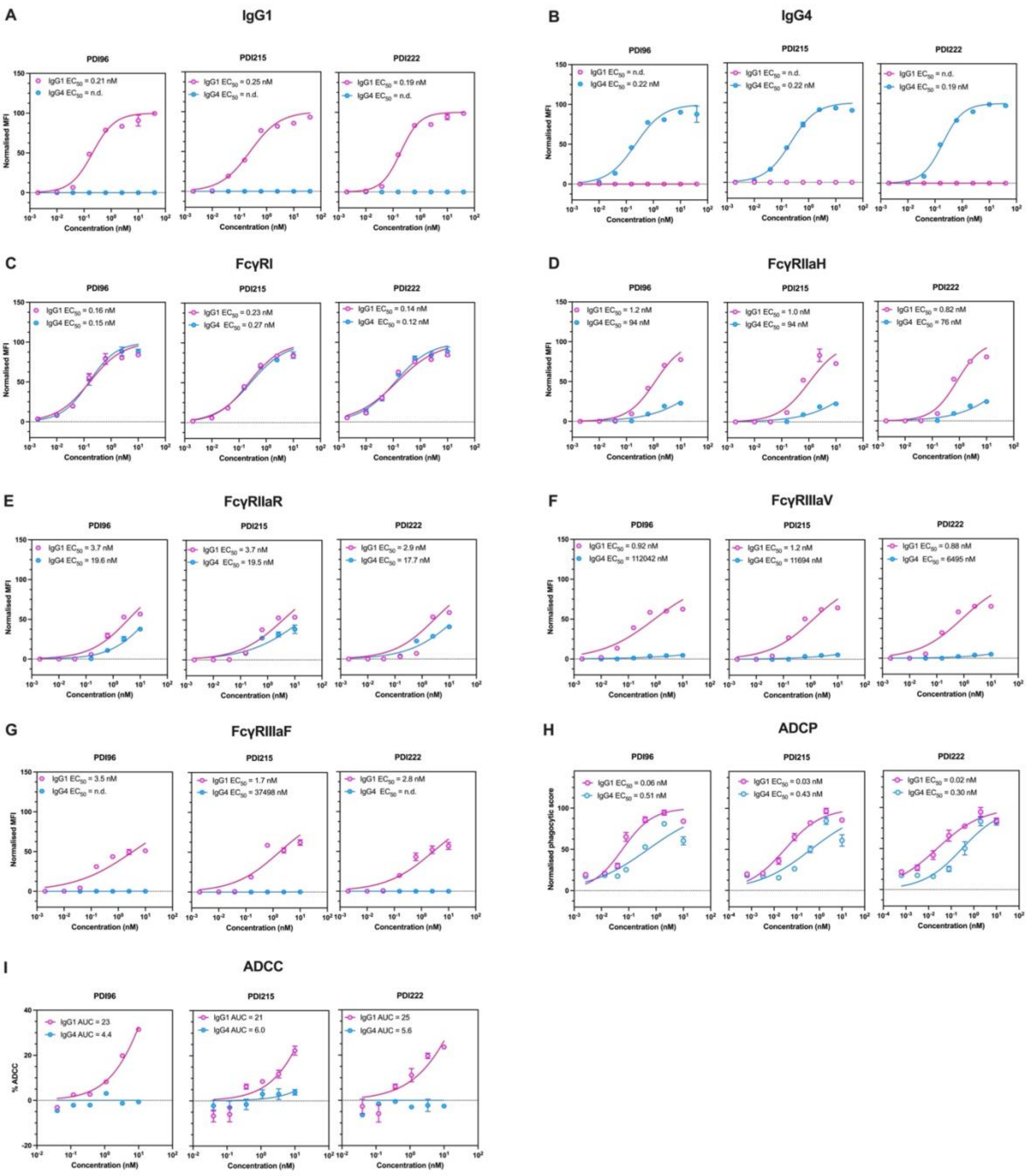

A
IgG1
PDI96
IgG1 EC50 = 0.21 nM
IgG4 EC50 = n.d.
PDI215
IgG1 EC50 = 0.25 nM
IgG4 EC50 = n.d.
PDI222
IgG1 EC50 = 0.19 nM
IgG4 EC50 = n.d.
B
IgG4
PDI96
IgG1 EC50 = n.d.
IgG4 EC50 = 0.22 nM
PDI215
IgG1 EC50 = n.d.
IgG4 EC50 = 0.22 nM
PDI222
IgG1 EC50 = n.d.
IgG4 EC50 = 0.19 nM
C
FcγRI
PDI96
IgG1 EC50 = 0.16 nM
IgG4 EC50 = 0.15 nM
PDI215
IgG1 EC50 = 0.23 nM
IgG4 EC50 = 0.27 nM
PDI222
IgG1 EC50 = 0.14 nM
IgG4 EC50 = 0.12 nM
D
FcγRIIaH
PDI96
IgG1 EC50 = 1.2 nM
IgG4 EC50 = 94 nM
PDI215
IgG1 EC50 = 1.0 nM
IgG4 EC50 = 94 nM
PDI222
IgG1 EC50 = 0.82 nM
IgG4 EC50 = 76 nM
E
FcγRIIaR
PDI96
IgG1 EC50 = 3.7 nM
IgG4 EC50 = 19.6 nM
PDI215
IgG1 EC50 = 3.7 nM
IgG4 EC50 = 19.5 nM
PDI222
IgG1 EC50 = 2.9 nM
IgG4 EC50 = 17.7 nM
F
FcγRIIIaV
PDI96
IgG1 EC50 = 0.92 nM
IgG4 EC50 = 112042 nM
PDI215
IgG1 EC50 = 1.2 nM
IgG4 EC50 = 11694 nM
PDI222
IgG1 EC50 = 0.88 nM
IgG4 EC50 = 6495 nM
G
FcγRIIIaF
PDI96
IgG1 EC50 = 3.5 nM
IgG4 EC50 = n.d.
PDI215
IgG1 EC50 = 1.7 nM
IgG4 EC50 = 37498 nM
PDI222
IgG1 EC50 = 2.8 nM
IgG4 EC50 = n.d.
H
ADCP
PDI96
IgG1 EC50 = 0.06 nM
IgG4 EC50 = 0.51 nM
PDI215
IgG1 EC50 = 0.03 nM
IgG4 EC50 = 0.43 nM
PDI222
IgG1 EC50 = 0.02 nM
IgG4 EC50 = 0.30 nM
I
ADCC
PDI96
IgG1 AUC = 23
IgG4 AUC = 4.4
PDI215
IgG1 AUC = 21
IgG4 AUC = 6.0
PDI222
IgG1 AUC = 25
IgG4 AUC = 5.6
Normalised MFI
Normalised phagocytic score
% ADCC
Concentration (nM)

Fig S3. Antigen binding and Fc-mediated responses of PDI monoclonal antibodies against ancestral SARS-CoV-2 spike trimer. Binding of PDI monoclonal antibodies (mAb) to ancestral Spike trimer as measured by anti-human **(A)** IgG1 or **(B)** IgG4 detection reagents via multiplex. **(C-G)** Binding of PDI IgG1 and IgG4 mAbs to soluble recombinant human FcγRs. The normalised median fluorescent intensity (MFI) is graphed. **(I)** Antibody-dependent cellular phagocytosis of ancestral spike-coated beads mediated by PDI IgG1 or IgG4 mAbs. The normalised phagocytic score is graphed. **(J)** Antibody-dependent cellular cytotoxicity of Ramos cells expressing full length ancestral spike trimer mediated by PDI IgG1 or IgG4 mAbs. The half-maximal binding concentration (EC_{50}) was calculated using a four-parameter nonlinear regression model. Error bars indicate the standard error of mean.

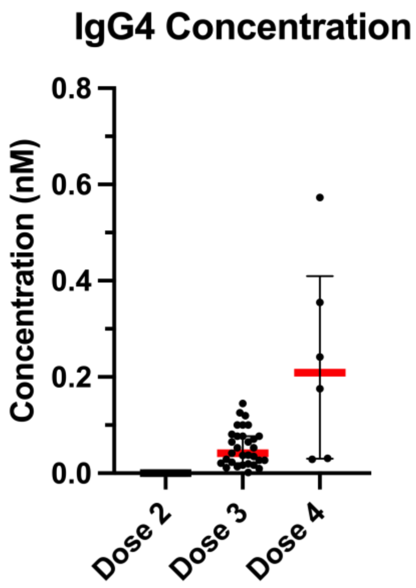

Fig S4. IgG4 Concentrations post mRNA Vaccination. IgG4 concentrations in plasma samples collected post dose two (n = 46), dose three (n = 31) and dose four (n = 6) of BNT162b2 vaccination. The red lines indicate the median concentration for each dose. Error bars indicate interquartile range.

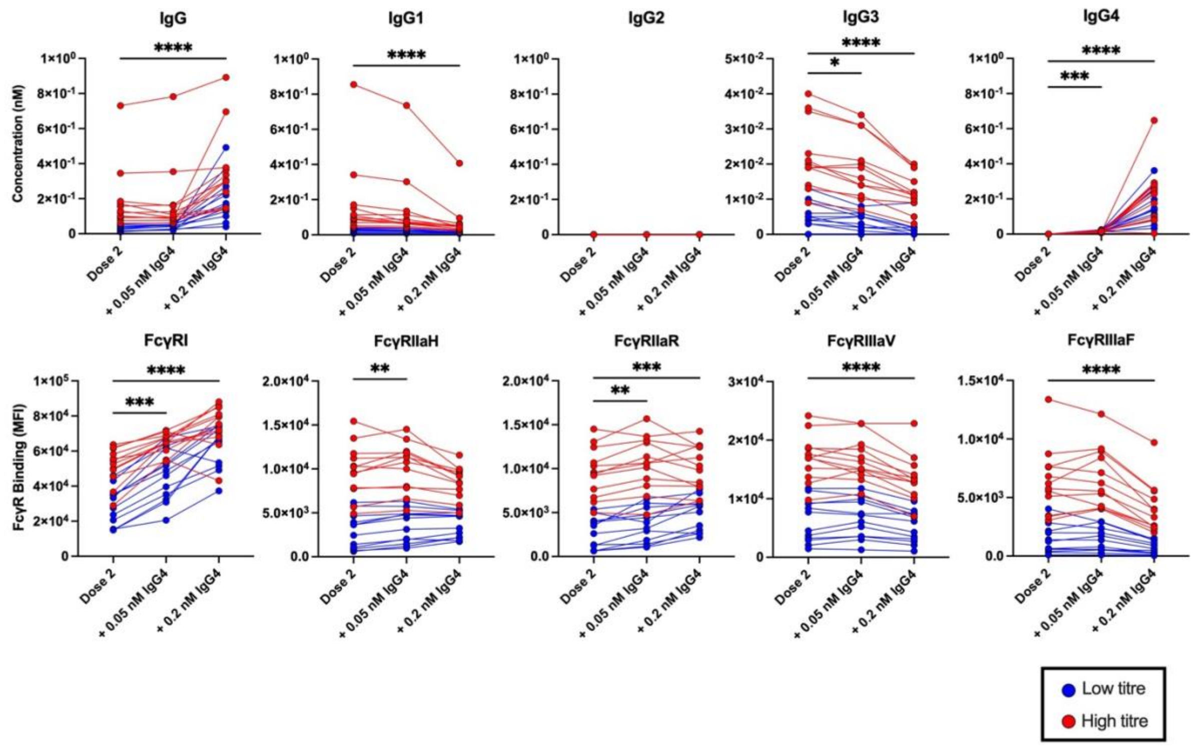

Fig S5. Change in IgG responses following IgG4 mAb spiking into post dose two BNT162b2 vaccinated plasma. Comparison of ancestral spike trimer-specific Pan-IgG, IgG1-4 titres and FcγR binding activity of plasma collected following two doses of BNT162b2 vaccination (n = 24, median 30 days post-vaccination) following addition of 0.05 nM or 0.2 nM of IgG4 SARS-CoV-2 monoclonal antibody (mAb) cocktail. Statistical significance was assessed using Friedman's test with Dunn's multiple comparison. * $p<0.05$, ** $p<0.01$, ***$p<0.001$, * $p<0.0001$. MFI = median fluorescent intensity.

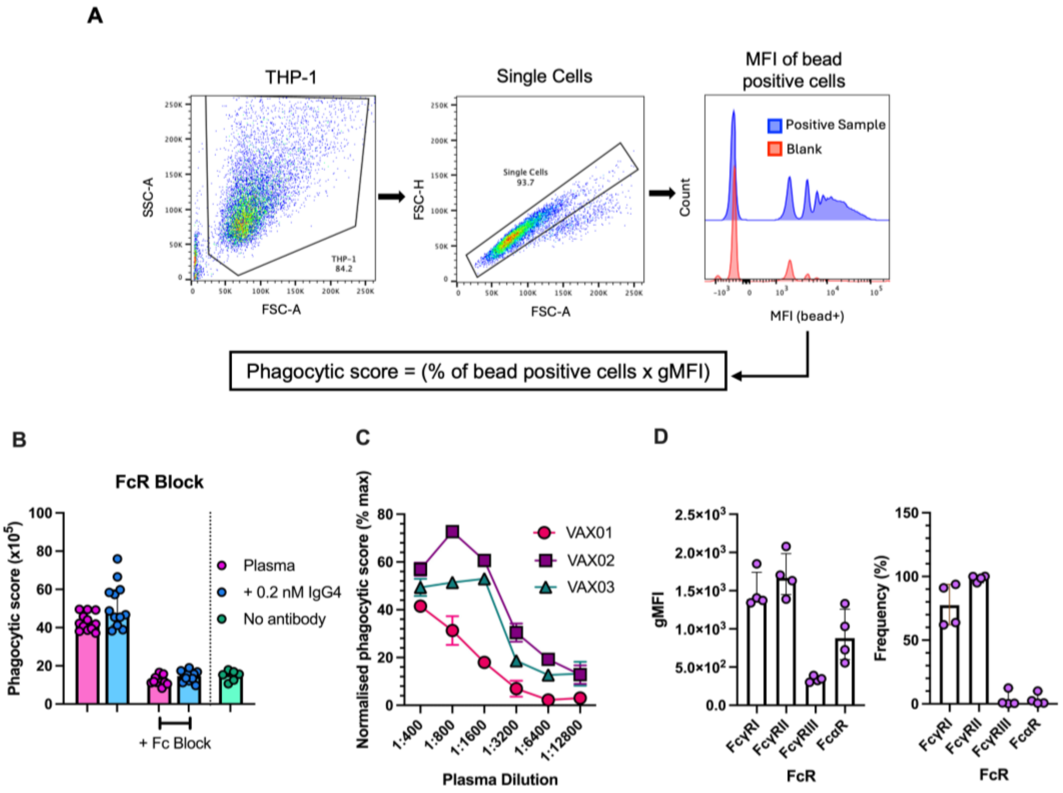

Fig S6. Gating strategy of ADCP bead-based assay and FcR expression of THP-1 monocytes. (A) The antibody dependent bead-based cellular phagocytosis assay (ADCP assay) gating strategy. First, THP-1 monocytes were gated, followed by single cells and bead positive cells. Finally, a phagocytic score was calculated. **(B)** Phagocytic score of plasma collected post second dose mRNA vaccination and post dose two plasma following 0.2 nM IgG4 monoclonal antibody addition in the presence or absence of an Fc block. **(C)** Serial dilution of a subset of post dose two plasma. A 1:3200 plasma dilution was chosen as the optimal dilution to avoid saturation at lower dilutions. **(D)** Expression of CD16 (FcγRIII), CD32 (FcγRII), CD64 (FcγRI) and CD89 (FcαR) on THP-1 monocytes**.**

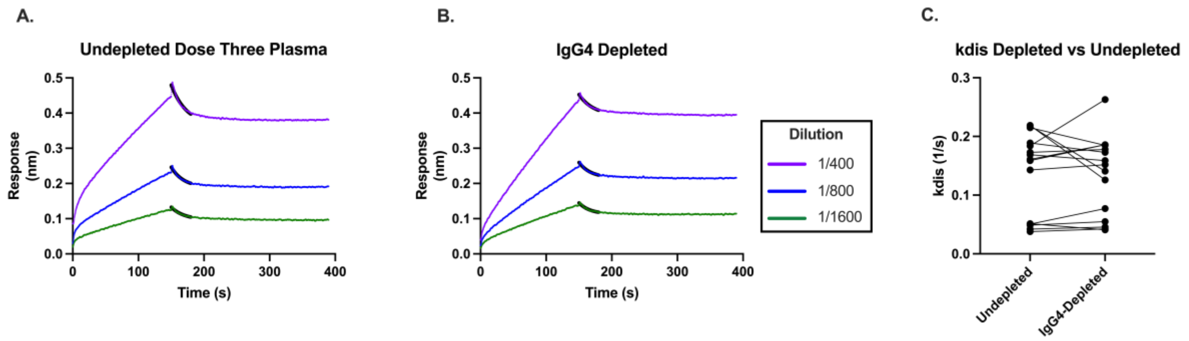

Fig S7 Binding Kinetics of IgG4-depleted and undepleted post dose three plasma. IgG4 was depleted from plasma samples collected one month post dose three BNT162b2 vaccination (n = 16, median 30 days post vaccination). Binding kinetics of plasma to ancestral SARS-CoV-2 spike trimer was assessed by biolayer interferometry (BLI). Representative BLI sensograms of interaction between immobilised ancestral spike trimer to **(A)** undepleted and **(B)** IgG4-depleted plasma. Plasma was diluted serially two-folds starting from 1 in 400. The curves were fitted based on the 1:1 binding kinetic model using data analysis software 12.0 (FortéBio). **(C)** Comparison of dissociation rate constant (kdis) of IgG4-depleted and undepleted plasma. The average kdis calculated at the three different dilutions of each condition was plotted.

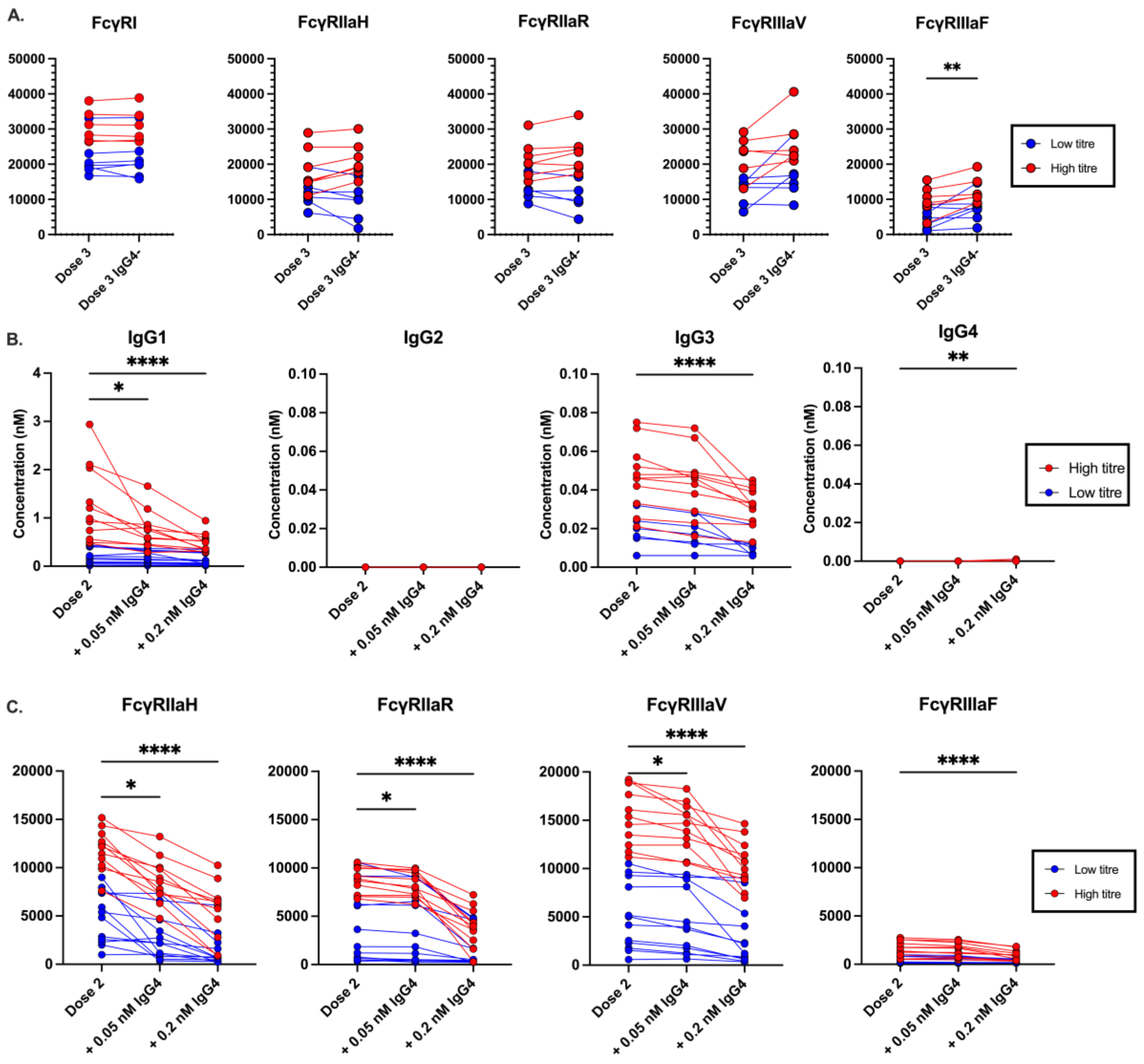

Fig S8. Change IgG responses following IgG4 depletion and (Fab')$_2$ spiking. (A) Comparison of ancestral spike trimer-specific FcγR binding activity of plasma collected post dose three BNT162b2 vaccination (n = 13, median 30 days post vaccination) and IgG4-depleted dose three plasma. **(B-C)** Comparison of ancestral spike trimer-specific **(B)** IgG1-4 titres and **(C)** FcγR binding activity of plasma collected following two doses of BNT162b2 vaccination (n = 24, median 30 days post-vaccination) following addition of 0.05 nM or 0.2 nM of the (Fab)2 fragments of IgG4 SARS-CoV-2 monoclonal antibodies. Statistical significance was assessed using Friedman's test with Dunn's multiple comparison. * p<0.05, ** p<0.01, **** p<0.0001.

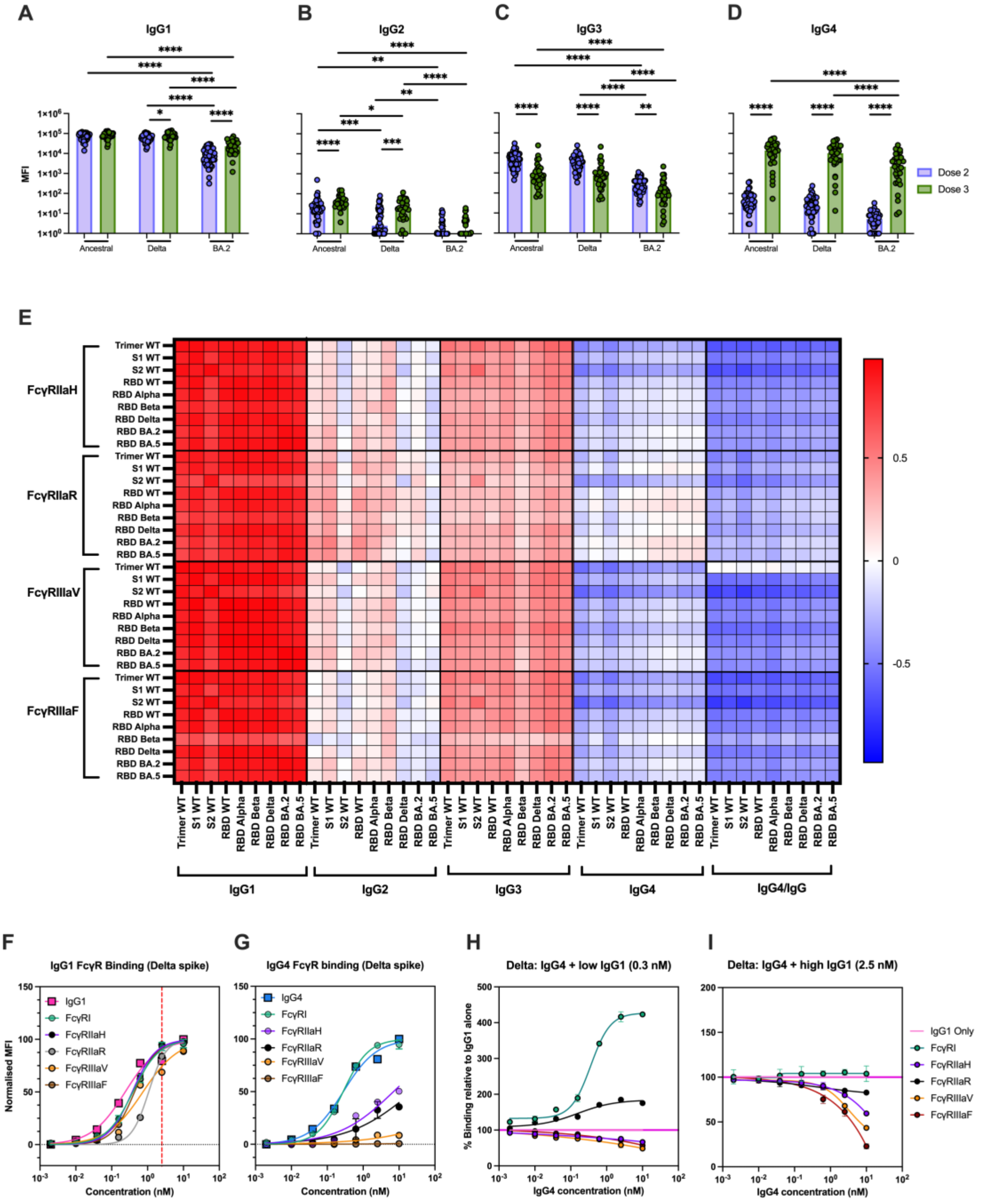
A
IgG1
B
IgG2
C
IgG3
D
IgG4
MFI
Ancestral
Delta
BA.2
Dose 2
Dose 3
E
FcγRIIaH
FcγRIIaR
FcγRIIIaV
FcγRIIIaF
Trimer WT
S1 WT
S2 WT
RBD WT
RBD Alpha
RBD Beta
RBD Delta
RBD BA.2
RBD BA.5
IgG1
IgG2
IgG3
IgG4
IgG4/IgG
0.5
0
-0.5
F
IgG1 FcγR Binding (Delta spike)
Normalised MFI
Concentration (nM)
IgG1
FcγRI
FcγRIIaH
FcγRIIaR
FcγRIIIaV
FcγRIIIaF
G
IgG4 FcγR binding (Delta spike)
IgG4
FcγRI
FcγRIIaH
FcγRIIaR
FcγRIIIaV
FcγRIIIaF
Concentration (nM)
H
Delta: IgG4 + low IgG1 (0.3 nM)
% Binding relative to IgG1 alone
IgG4 concentration (nM)
I
Delta: IgG4 + high IgG1 (2.5 nM)
IgG4 concentration (nM)
IgG1 Only
FcγRI
FcγRIIaH
FcγRIIaR
FcγRIIIaV
FcγRIIIaF

Fig S9. IgG responses against SARS-CoV-2 variants. (A-D) Plasma IgG1-4 the receptor binding domain (RBD) from the ancestral, Delta and Omicron BA.2 strains following two and three doses of BNT162b2 vaccination. Statistical significance between doses and variants was assessed using Mann-Whitney *U*-test and Kruskall Wallis test with Dunn's multiple comparison respectively. **(E)** Spearman correlation between IgG1-4 titres and IgG4:IgG ratio with FcγR binding against ancestral and variant SARS-CoV-2 antigens. Binding of **(F)** IgG1 and **(G)** IgG4 mAb cocktails to IgG1 or IgG4 detection reagents or recombinant soluble FcγRs against Delta spike trimer. The normalised median fluorescent intensity (MFI) of each detection reagent was plotted. **(H-I)** FcγR binding activity of IgG4 mAb cocktail was titrated into IgG1 mAb cocktail and binding to FcγRs against Delta spike. Error bars indicate SEM. Curves were fitted using a four-parameter nonlinear regression. * $p<0.05$, ** $p<0.01$****$p<0.0001$.

SUPPLEMENTARY TABLES

Table S1. Summary of cohort demographics.

	Dose 2 (n = 46)	Dose 3 (n = 31)
**Age, median (IQR), years**	34 (29-46)	34 (29-47)
**Gender**		
**Female, *n* (%)**	30 (65%)	22 (71%)
**Male, *n* (%)**	16 (35%)	9 (29%)
**Time between vaccination to sample collection, median (IQR), days**	30 (13-30)	30 (14-39)

Table S2. Binding kinetics of anti-SARS-CoV-2 monoclonal antibodies.

	K_D (M)	kon (1/Ms)	Kdis (1/s)
**RBD WT**			
PDI96 IgG1	2.73E-09	2.13E+05	5.75E-04
PDI96 IgG4	2.27E-09	2.28E+05	5.17E-04
PDI215 IgG1	2.20E-10	8.91E+05	1.96E-04
PDI215 IgG1	2.17E-10	5.76E+05	1.25E-04
PDI222 IgG1	1.96E-10	2.53E+05	5.97E-05
PDI222 IgG4	2.20E-10	2.44E+05	5.37E-05
**RBD Delta**			
PDI96 IgG1	3.64E-09	1.30E+05	4.69E-04
PDI96 IgG4	2.70E-09	1.22E+05	3.31E-04
PDI215 IgG1	3.31E-10	3.37E+05	1.10E-04
PDI215 IgG1	3.16E-10	4.36E+05	1.57E-04
PDI222 IgG1	<1.0E-12	3.47E+05	<1.0E-07
PDI222 IgG4	<1.0E-12	3.95E+05	<1.0E-07
**RBD BA.2**			
PDI96 IgG1	n.d.	n.d.	n.d.
PDI96 IgG4	n.d.	n.d.	n.d.
PDI215 IgG1	3.36E-10	6.18E+05	2.07E-04
PDI215 IgG1	2.10E-10	8.17E+05	1.71E-04
PDI222 IgG1	2.07E-09	7.64E+05	1.58E-03
PDI222 IgG4	2.03E-09	1.04E+06	2.13E-03
**FcγRI**			
PDI96 IgG1	<1.0E-12	8.15E+05	<1.0E-07
PDI96 IgG4	7.40E-09	2.96E+05	2.20E-03
PDI215 IgG1	<1.0E-12	4.72E+05	<1.0E-07
PDI215 IgG1	3.47E-09	7.71E+05	2.39E-03
PDI222 IgG1	4.90E-11	7.13E+05	3.49E-05
PDI222 IgG4	1.73E-09	6.85E+05	1.18E-03
**FcγRIIa-H131**			
PDI96 IgG1	1.56E-07	5.99E+04	9.37E-03
PDI96 IgG4	2.80E-04	3.82E+03	1.07E-01
PDI215 IgG1	2.36E-07	4.03E+04	9.46E-03
PDI215 IgG4	2.01E-04	1.95E+03	3.91E-01
PDI222 IgG1	2.56E-07	2.65E+04	6.80E-03
PDI222 IgG4	3.58E-04	7.49E+03	2.67E-01
**FcγRIIa-R131**			
PDI96 IgG1	1.00E-07	2.18+05	2.19E-02

PDI96 IgG4	1.87E-05	2.44E+04	1.05E-01
PDI215 IgG1	1.44E-07	1.03E+05	1.46E-02
PDI215 IgG4	1.89E-06	5.95E+04	1.12E-01
PDI222 IgG1	2.51E-07	1.49E+05	2.72E-02
PDI222 IgG4	2.56E-06	8.63E+04	2.21E-01
**FcγRIIIa-V158**			
PDI96 IgG1	1.16E-07	6.34E+04	7.34E-03
PDI96 IgG4	n.d.	n.d.	n.d.
PDI215 IgG1	5.71E-07	3.59E+04	2.05E-02
PDI215 IgG4	n.d.	n.d.	n.d.
PDI222 IgG1	1.12E-07	1.90E+04	2.14E-02
PDI222 IgG4	n.d.	n.d.	n.d.
**FcγRIIIa-F158**			
PDI96 IgG1	1.04E-06	5.48E+04	5.70E-02
PDI96 IgG4	n.d.	n.d.	n.d.
PDI215 IgG1	3.94E-06	7.00E+03	3.58E-02
PDI215 IgG4	n.d.	n.d.	n.d.
PDI222 IgG1	1.08E-06	5.71E+04	5.72E-02
PDI222 IgG4	n.d.	n.d.	n.d.

Table S3. EC_{50} of IgG1 and IgG4 monoclonal antibody cocktails.

	Ancestral Trimer EC_{50} (nM)		Delta Trimer EC_{50} (nM)		BA.2 Trimer EC_{50} (nM)	
	IgG1	IgG4	IgG1	IgG4	IgG1	IgG4
IgG1	0.16	n.d.	0.23	n.d.	0.50	n.d.
IgG4	n.d.	0.19	n.d.	0.28	n.d.	0.49
FcγRI	0.39	0.30	0.39	0.28	1.21	0.74
FcγRIIa-H131	0.49	19.3	0.38	23.6	0.65	8.93
FcγRIIa-R131	0.43	4.3	1.07	7.00	1.69	11.1
FcγRIIIa-V158	0.48	2081	0.74	2792	1.21	666
FcγRIIIa-F158	0.42	985260	0.43	622105	1.53	1052
ADCP	0.009	0.044				
ADCC (area under curve)	63.6	9.9				

Table S4. List of antigens used for multiplex assay.

Protein	Source	Catalogue No.
SARS-CoV-2 (2019-nCoV) Spike S1+S2 trimer	SinoBiological	40589-V08H4
SARS-CoV-2 B.1.351 Spike S1+S2 trimer	SinoBiological	40589-V08H13
SARS-CoV-2 B.1.617 Spike S1+S2 trimer	SinoBiological	40589-V08H10
SARS-CoV-2 BA.2 Spike S1+S2 trimer	SinoBiological	40589-V08H28
SARS-CoV-2 (BA.4/BA.5/BA.5.2) Spike S1+S2 trimer	SinoBiological	40589-V08H32
SARS-CoV-2 (2019-nCoV) Spike RBD	SinoBiological	40592-V08H
Beta SARS-CoV-2 Spike RBD	SinoBiological	40592-V08H85
Delta SARS-CoV-2 Spike RBD	SinoBiological	40592-V08H90
SARS-CoV-2 B.1.1529 sub lineage BA.2 Spike RBD	SinoBiological	40592-V08H123
SARS-CoV-2 (BA.4/BA.5/BA.5.2) Spike RBD	SinoBiological	40592-V08H130
SARS-CoV-2 RBD Alpha – N501Y	WEHI	
SARS-CoV-2 RBD Beta – K417N, E484K, N501Y	WEHI	
SARS-CoV-2 RBD Delta – L452R, T478K	WEHI	
SARS-CoV-2 Ancestral Spike Trimer	Adam Wheatley	
SARS-CoV-2 (2019-nCoV) Spike S1	SinoBiological	40591-V08H
SARS-CoV-2 Ancestral Spike S2	Adam Wheatley	
SIV gp120	SinoBiological	40415-V08H
Influenza A H1N1 (A/Cal/07/2009)	SinoBiological	11085-V08H

Table S5. List of detection antibodies used.

Antibody	Clone	Company	Catalogue No.
Mouse anti-human IgG1, biotinylated	MTG1218	Mabtech	3851-14-250
Mouse anti-human IgG2, biotinylated	HP6200	Mabtech	3852-6-250
Mouse anti-human IgG3, biotinylated	MTG34	Mabtech	3853-6-250
Mouse anti-human IgG4, biotinylated	MTG42	Mabtech	3854-6-250
Mouse anti-human total IgG1, biotinylated	MTG78/145	Mabtech	3850-6-250
Mouse anti-human IgG1 PE	HP60001	Southern Biotech	9054-09
Mouse anti-human IgG4 PE	HP6025	Southern Biotech	9200-09
Mouse anti-human CD16 BV605	3G8	BioLegend	302040
Mouse anti-human CD32 FITC	FUN-2	BioLegend	303204
Mouse anti-human CD64 BV510	10.1	BioLegend	305028
Mouse anti-human CD89 APC	A59	BioLegend	354106

Table S6: Parameters for *in silico* FcγR ODE models.

Parameter	Value	Unit	Source
K_D: IgG-Trimer	0.22	nM	BLI Measurements
K_D: IgG1-FcγRIIaH	192	nM	(*11*)
K_D: IgG2-FcγRIIaH	2222	nM	(*11*)
K_D: IgG3-FcγRIIaH	1124	nM	(*11*)
K_D: IgG4-FcγRIIaH	5582	nM	(*11*)
K_D: IgG1-FcγRIIaR	286	nM	(*11*)
K_D: IgG2-FcγRIIaR	10000	nM	(*11*)
K_D: IgG3-FcγRIIaR	1099	nM	(*11*)
K_D: IgG4-FcγRIIaR	4762	nM	(*11*)
Trimer Initial Concentration	2.5	nM	Multiplex Assay Parameter
FcγRIIa Initial Concentration	20	nM	Multiplex Assay Parameter
